# Extensive nuclear gyration and pervasive non-genic transcription during primordial germ cell development in zebrafish

**DOI:** 10.1242/dev.193060

**Published:** 2021-01-20

**Authors:** Stefan Redl, Antonio M. de Jesus Domingues, Edoardo Caspani, Stefanie Möckel, Willi Salvenmoser, Maria Mendez-Lago, René F. Ketting

**Affiliations:** 1 Biology of Non-coding RNA Group, Institute of Molecular Biology, Ackermannweg 4, 55128 Mainz, Germany; 2 International PhD Programme on Gene Regulation, Epigenetics & Genome Stability, 55128 Mainz, Germany; 3 Flow Cytometry Core Facility, Institute of Molecular Biology (IMB), Ackermannweg 4, 55128 Mainz, Germany; 4 Institute of Zoology, Evolution and Developmental Biology, University of Innsbruck, Technikerstraβe 25, 6020 Innsbruck, Austria; 5 Genomics Core Facility, Institute of Molecular Biology (IMB), Ackermannweg 4, 55128 Mainz, Germany; 6 Institute of Developmental Biology and Neurobiology, Johannes Gutenberg University, 55099 Mainz, Germany

**Keywords:** Zebrafish, Primordial germ cell, Zygotic activation, piRNA, Intergenic transcription, Nuclear morphology, Gyration, Nuage

## Abstract

Primordial germ cells (PGCs) are the precursors of germ cells, which migrate to the genital ridge during early development. Relatively little is known about PGCs after their migration. We studied this post-migratory stage using microscopy and sequencing techniques, and found that many PGC-specific genes, including genes known to induce PGC fate in the mouse, are only activated several days after migration. At this same time point, PGC nuclei become extremely gyrated, displaying general broad opening of chromatin and high levels of intergenic transcription. This is accompanied by changes in nuage morphology, expression of large loci (PGC-expressed non-coding RNA loci, PERLs) that are enriched for retro-transposons and piRNAs, and a rise in piRNA biogenesis signatures. Interestingly, no nuclear Piwi protein could be detected at any time point, indicating that the zebrafish piRNA pathway is fully cytoplasmic. Our data show that the post-migratory stage of zebrafish PGCs holds many cues to both germ cell fate establishment and piRNA pathway activation.

## Abstract (precis)

**Highlighted Article:** In contrast to common thinking, zebrafish primordial germ cells do not rest after arrival at the genital ridge, but display many morphological changes accompanied by pervasive intergenic transcription and piRNA activation.

## INTRODUCTION

The specification of the primordial germ cells (PGCs) in the zebrafish starts at the 32-cell stage by the uptake of germ plasm, or nuage, an electron-dense phase-separated structure containing RNA and protein, into four blastomeres (Raz, 2003). The germ plasm-containing blastomeres initially divide asymmetrically and only daughter cells that inherit the germ plasm can at later stages become a germ cell (Knaut et al., 2000). At sphere stage, the germ plasm disperses into smaller granules and associates increasingly with the nuclear membrane. From that point onwards, both daughter cells inherit germ plasm during cell divisions, and thereby the potential for becoming germ cells.

While the embryo develops, the PGCs divide and migrate towards the genital ridge, where they arrive around 16-24 h post- fertilization (hpf) (Raz, 2003; Weidinger et al., 1999; Yoon et al., 1997). By this time, the number of PGCs has increased to 25-50 (Braat et al., 1999; Knaut et al., 2000; Weidinger et al., 1999; Yoon et al., 1997). Even though the initial specification and migration of PGCs in zebrafish is well studied, little is known about what happens to these cells from after their arrival at the genital ridge, until the time they start to form a definite gonad, which happens around 10 days post fertilization (dpf) (Leerberg et al., 2017; Tzung et al., 2014). The number of PGCs stays constant in this period, indicating these cells are not proliferating (Tzung et al., 2014). Furthermore, it was recently shown that global DNA methylation is maintained during this period, indicating that no de-methylated state occurs during the zebrafish germ cell development cycle (Ortega-Recalde et al., 2019).

The piRNA pathway is an RNAi-related pathway, found in the germ cells of many animals. Argonaute proteins are characteristic of all RNAi-related pathways, and represent the functional core of such pathways, by binding the small RNA co-factors and triggering gene-regulatory effects. PiRNAs are the small RNA co-factors of a distinct branch of argonaute proteins, known as Piwi proteins. The zebrafish genome encodes two Piwi proteins: Ziwi and Zili (Houwing et al., 2007, 2008). Ziwi mainly binds piRNAs that are antisense with respect to the mRNA of their targets, which are mostly transposable elements (TEs), and are characterized by a strong bias for a 5′ uracil. In contrast, Zili binds mostly sense-piRNAs, which are enriched for an adenosine at position 10. Biogenesis of Zili-bound piRNAs is triggered by Ziwi-mediated cleavage of a target RNA and vice versa. These relationships lead to a characteristic enrichment of 10-nucleotide 5′ overlaps between Ziwi- and Zili-bound piRNAs (ping-pong signature). In addition to this type of Piwi-directed piRNA biogenesis, another endonuclease, Zucchini (Zuc), also named Pld6, has been implicated in generating piRNAs (Han et al., 2015; Ipsaro et al., 2012; Mohn et al., 2015). Although not yet directly studied in zebrafish, piRNA sequence characteristics strongly suggest that Pld6 also acts in zebrafish to produce piRNAs (Gainetdinov et al., 2018).

Ziwi and its associated piRNAs are maternally provided via the germ plasm, and are thought to provide a first line of defense against active transposon mRNAs in the zygote (Houwing et al., 2007). Maternal priming of the Piwi pathway has been observed also in *Drosophila* (Brennecke et al., 2008) and *C. elegans* (Luteijn et al., 2012; Shirayama et al., 2012), and, in ciliates, parental small RNAs guide the genome rearrangements of newly established zygotes (Lepere et al., 2008). These studies indicate that maternal initiation of small RNA pathways is a widely conserved phenomenon. Interestingly, maternally provided Ziwi protein can be detected in germ cells for up to 3 weeks post-fertilization (Houwing et al., 2007), and it was postulated that this pool may prime the zygotic piRNA pathway. *Ziwi* mutant germ cells are lost through apoptosis, starting at 20 dpf (Houwing et al., 2007). The second PIWI protein, Zili, is not transmitted maternally, and only starts to be expressed zygotically around 3 dpf (Houwing et al., 2008). Although it first seems to be evenly distributed throughout the PGCs, and is only excluded from DAPI-dense regions, its distribution becomes increasingly restricted to perinuclear granules (Houwing et al., 2008). These observations suggested that Zili may initially drive a nuclear piRNA pathway in the zebrafish, followed by restriction to the cytoplasm at later stages (Houwing et al., 2008). *Zili* mutant PGCs appear to arrest, but not to die, at a stage resembling PGCs at 3 dpf, the time when Zili should start to be expressed (Houwing et al., 2007, 2008). This difference between *zili* and *ziwi* mutant PGCs is consistent with the idea that maternally provided Ziwi requires zygotically expressed Zili to sustain PGC development.

Biogenesis of piRNA happens within an electron-dense structure called nuage. These structures represent phase-separated condensates driven by both RNA and protein molecules. Nuage is found in germ cells of many animals, including mammals. Interestingly, different types of nuage have been described, based on different appearances under electron microscopy or on the absence or presence of specific proteins (Aravin et al., 2009; Wan et al., 2018). Nuage in adult zebrafish germ cells is known to be very electron dense and tightly associated with mitochondria, whereas nuage in early embryos is more diffuse and less associated with mitochondria (Herpin et al., 2007; Huang et al., 2011). Proteins known to be required for normal nuage formation in zebrafish are Tdrd1 (Huang et al., 2011) and Tdrd6a (Roovers et al., 2018).

In an effort to better interpret mutant phenotypes in the zebrafish piRNA pathway, we set out to study the development of PGCs between 1 and 10 dpf, by sequencing small RNAs, mRNAs and total RNA populations, and with immunofluorescence and electron microscopy. Our results not only revealed PGC-Expressed non-coding RNA loci, or PERLs, as likely sources of piRNA precursor transcripts during the zygotic activation of the piRNA pathway, but also revealed that germ cell fate establishment may not take place until several days following arrival of the PGCs at the gonadal ridge. In addition, our work provides useful resources for future studies on zebrafish germ cell development.

## RESULTS

### Heavily gyrated PGC nuclei at 3 dpf

**Fig. 1.**
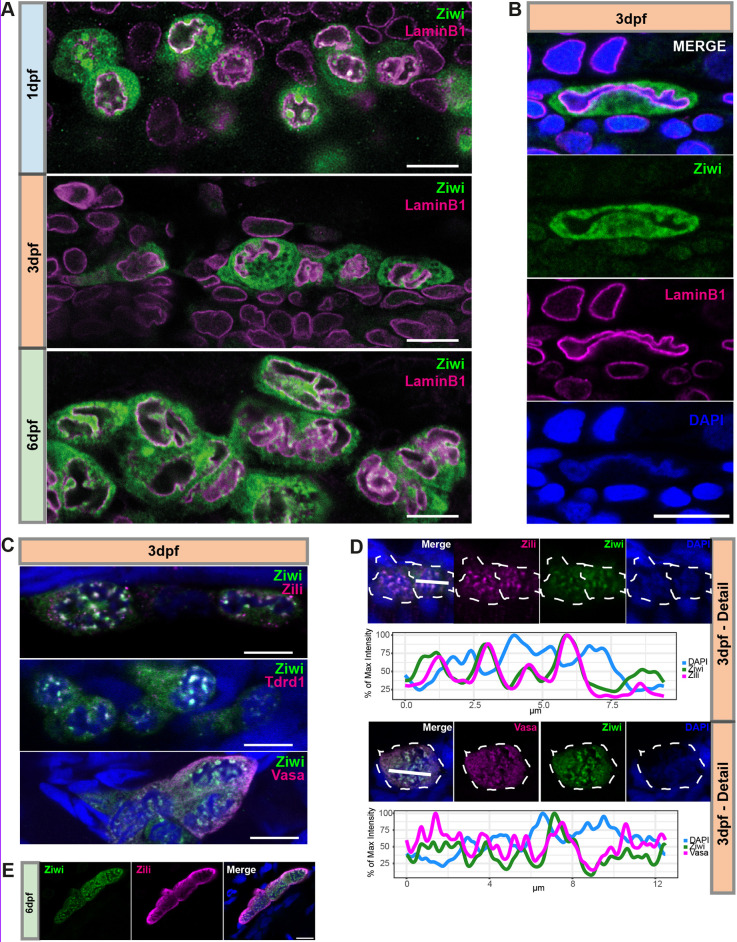
**piRNA pathway components localize outside of gyrated nuclei.** (A) Immunostaining for Ziwi (green) and LaminB1 (magenta) in PGCs at indicated time points. Scale bars: 10 µm. (B) A 3 dpf PGC with Ziwi (green), LaminB1 (magenta) and DAPI (blue). Scale bar: 10 µm. (C) Double immunostaining for colocalization of Ziwi and Zili, Tdrd1 and Vasa in PGCs at 3 dpf. Scale bars: 10 µm. (D) Colocalization analysis of Zili and Ziwi, and Ziwi and Vasa. Germ cells are indicated by the white dashed line. Blue staining is DAPI. Line plot of indicated selection for Ziwi and Zili (top) and Ziwi and Vasa (bottom) with DAPI. *x*-axis indicates the position along the indicated bar from left to right in µm; *y*-axis indicates percentage of maximum intensity. (E) Immunostaining of Ziwi and Zili at 6 dpf. Blue staining is DAPI. Scale bar: 10 µm.

The localization of the piRNA-pathway associated proteins Ziwi, Zili and Tdrd1 has already been reported for 3 dpf and 7 dpf by our group. At the time, we concluded that Ziwi and Tdrd1 localize to perinuclear granules at these time points, whereas Zili is both in the cytoplasm as well as within the nucleus at 3-7 dpf, but becomes restricted to perinuclear granules at later stages (Houwing et al., 2008). It was already noted that Zili was excluded from DAPI bright spots (Houwing et al., 2007, 2008; Huang et al., 2011). When we repeated these stainings, we noticed that it was extremely difficult to discern nucleus from cytoplasm at these stages. We therefore decided to use a LaminB1 antibody to stain for the nuclear envelope and combined it with staining for Ziwi (Fig. 1A). At 1 dpf, PGCs nuclei were surrounded by large Ziwi-positive granules, and had no or, at most, one major invagination (Fig. 1A). However, at 3 and 6 dpf, we found the nuclear envelope to be heavily gyrated, forming very thin extensions (Fig. 1A,B). Ziwi had a very granular (Fig. 1C) or more diffuse distribution throughout the cytoplasm (Fig. 1A,B). Regardless of this, Ziwi always localized outside the nucleus (Fig. 1A,B).

This novel, peculiar nuclear morphology raised the possibility that the previously described nuclear localization of Zili (Houwing et al., 2008) in fact represented cytoplasmic pockets that are seemingly found inside the nuclei in optical sections. Unfortunately, the antibody-sources prevented direct LaminB1-Zili co-stainings to assess Zili localization. Hence, we combined Zili staining with DAPI and Ziwi to visualize both cytoplasm and chromatin at 3 dpf (Fig. 1C,D), when Zili starts to be expressed, and 6 dpf (Fig. 1E). This revealed good overlap between Ziwi and Zili, strongly suggesting that Zili is also cytoplasmic. Very similar outcomes were found for two additional piRNA pathway components, both known to be exclusively cytoplasmic: Vasa and Tdrd1 (Fig. 1C,D). We conclude that both Ziwi and Zili localize to the cytoplasm of PGCs, and that, starting at 3 dpf, PGC nuclei become heavily gyrated, resulting in large cytoplasmic intrusions into the nuclei.

### Electron microscopy reveals two types of nuage

**Fig. 2.**
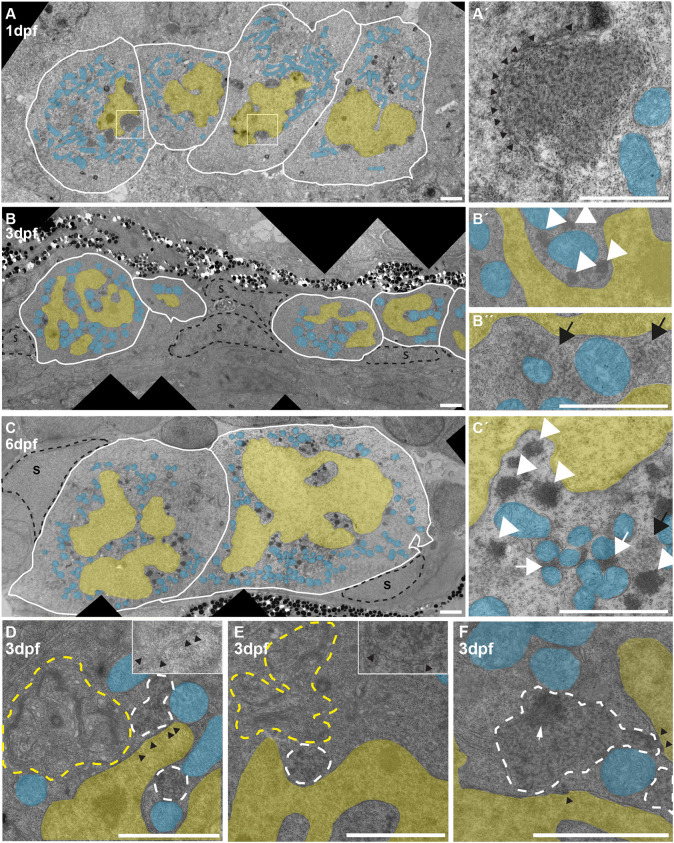
**Electron microscopy of wild-type PGCs.** (A) PGCs at 1 dpf. Nuclei (yellow) sometimes show one prominent invagination. Nuage can be seen as perinuclear dark patches. (A′) More-detailed view of one nuage patch, showing the granular texture. Black arrowheads indicate nuclear pores, visible as dark stretches within or interrupting the nuclear envelope. (B) PGCs at 3 dpf. The nuclei have acquired an extremely irregular outline and nuage is granular (black arrow, detail in B″) or forms dense granules (white arrowheads, detail in B′) around the nuclei. (C) PGCs at 6 dpf. The nuclei are still heavily gyrated (C′ shows C in more detail). Nuage is mostly very dense, and is found close to the nuclear envelope and in between clusters of mitochondria (white arrowheads and white arrows, respectively). Black arrow indicates granular nuage; S, somatic cells that contact PGCs extensively. (D,E) Two examples of 3 dpf PGCs in which granular nuage (white dashed outline) is in contact with nuclear pores (black arrowheads) and organelles, in this case mitochondria and Golgi (yellow outline). Insets show details without overlays. (F) An example of 3 dpf granular nuage with a more compacted part. Scale bars: 2 µm. Blue overlay indicates mitochondria; yellow overlay indicates nucleus.

To examine the observed nuclear morphology and other PGC characteristics in more detail, we turned to electron microscopy (EM). PGCs at 1 dpf could be found in close proximity to the yolk syncytial layer (ysl), as reported previously (Braat et al., 1999). Interestingly, they did not contact the ysl directly but were separated from it by cellular extensions of somatic cells (Fig. S1A). At all time points we observed that PGCs are closely associated with and surrounded by somatic cells (Fig. S1A-C). The nuclei of 1 dpf PGCs were mostly round with indentations found at the location of cytoplasmic nuage and sometimes showed a horseshoe shape, a characteristic also described for mouse PGCs (Clark and Eddy, 1975). Additionally, in agreement with previous reports in zebrafish (Huang et al., 2011) and *C. elegans* (Updike et al., 2011), nuclear pores were often detected close to nuage (Fig. 2A′). In contrast to nuage found in adult germ cells, nuage of PGCs at 1 dpf had a rather granular appearance (Fig. 2A,A′). Therefore, we will from here on refer to this type of nuage as ‘granular nuage’.

PGCs at 3 dpf were located adjacent to the pro-nephros (allentois) and a layer of pigment cells. Consistent with what we observed in immune fluorescence (Fig. 1A,B), PGCs had an unusually gyrated nucleus at 3 dpf (Fig. 2B). Nuclear invaginations were abundant, and cytoplasmic islands could be observed within nuclei in virtually any section. The nuclear membrane, however, was always intact. In addition, two types of nuage could be found. Some cells contained the granular nuage that we also observed in 1 dpf PGCs (Fig. 2B″). This type of nuage appeared spread out, and was not only associated with nuclear pores but often also made contact with organelles such as mitochondria and Golgi (Fig. 2D and E). The second type of nuage in 3 dpf PGCs was more electron dense and more compact than the granular nuage (Fig. 2B′), resembling nuage as it is typically found in adult germ cells. We mostly detected one type of nuage per cell, suggesting these two types of nuage may reflect different developmental stages. In a few cells, we could see nuage with both a granular and a dense area, possibly representing nuage in the process of switching from ‘granular’ to more compact nuage (Fig. 2F). These data reveal that structural changes in nuage occur around 3 dpf.

At 6 dpf, PGC nuclei remained gyrated. The nucleoplasm contained very few heterochromatic (dark) patches and appeared more euchromatic compared with those observed in 3 dpf PGCs. We quantified chromatin density using an approach that was recently published (Laue et al., 2019) and found that the level of heterochromatin at 6 dpf was significantly lower than at 1 and 3 dpf (Fig. S1D,E). This suggests that chromatin globally becomes more open at this time point. Nuage of PGCs at 6 dpf could be found in numerous, uniformly dark staining patches all along the nuclear envelope (Fig. 2C), very similar in appearance to nuage found in adult germ cells. Additionally, nuage in between mitochondria, detached from the nuclear membrane, was also abundant. Such structures have been named inter-mitochondrial cement in mammals (Eddy, 1974; Fawcett et al., 1970), but it is not clear whether these are functionally distinct from nuage at the nuclear periphery. We note that some factors in the piRNA pathway, such as Zucchini, are associated with the mitochondrial membrane (Ipsaro et al., 2012; Nishimasu et al., 2012), and it seems likely that the mitochondria-nuage interactions are related to specific steps in the piRNA pathway involving these proteins.

We conclude that PGCs undergo many morphological changes between 1 and 6 dpf. Next, we tested whether the piRNA pathway was required for the observed changes in nuage structure.

### Transition of granular to compact nuage requires zygotic Tdrd1 and Zili

The Tudor proteins Tdrd1 and Tdrd6a have been shown to be necessary for either nuage or proper germ plasm formation in zebrafish (Huang et al., 2011; Roovers et al., 2018). In *tdrd6a* mutants, embryonic germ plasm is affected, but nuage seems unaffected (Roovers et al., 2018). Here, we probed the presence and localization of Tdrd6a in 1, 3, 6 and 10 dpf PGCs (Fig. S1F). Tdrd6a colocalized with Ziwi in large perinuclear nuage granules at 1 dpf, but in 3 dpf PGCs was found either colocalizing with Ziwi in perinuclear nuage or was absent from PGCs (asterisk in Fig. S1F, 3 dpf). At 6 dpf, Tdrd6a protein was no longer detected in PGCs, and only reappeared in some germ cells at 10 dpf (Fig. S1F). We hypothesize that cells with Tdrd6a correspond to cells displaying granular nuage in EM, and those lacking Tdrd6a correspond to cells displaying adult-type nuage.

**Fig. 3.**
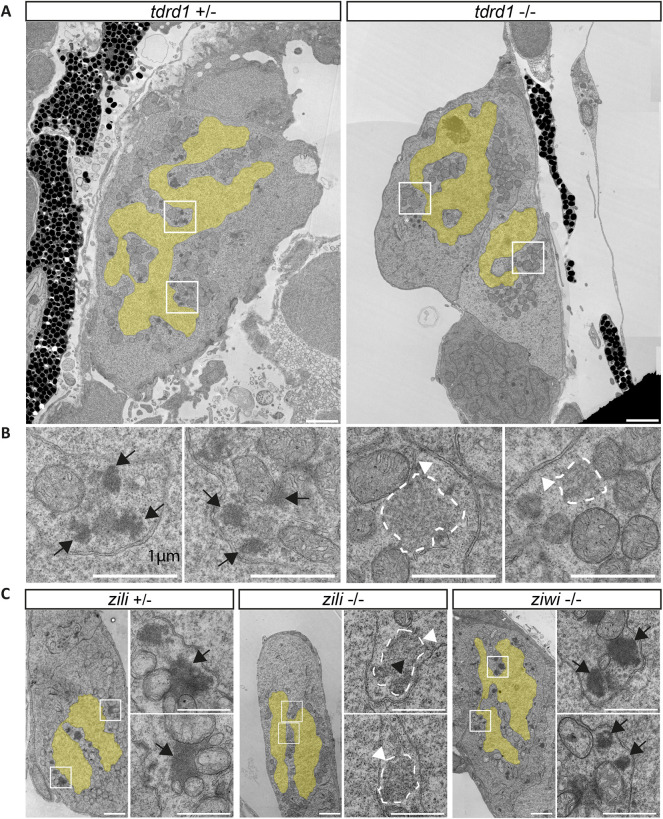
**Electron microscopy of mutant PGCs.** (A) Wild-type and *tdrd1* mutant PGCs at 6 dpf. Nuclei are overlaid with a yellow shade. The regions indicated by squares are shown in more detail in B. Asterisk indicates multi-vesicular-bodies, not compact nuage. Scale bars: 2 µm. (B) Detail of nuage structures. Only granular nuage is detectable (arrowhead and white dashed line) at 6 dpf in *tdrd1* mutants. The normal nuage in the wild type is indicated by black arrows. Scale bars: 1 µm. (C) PGCs at 6 dpf from *zili* heterozygous animal and from *zili* and *ziwi* mutants. Right panels for each genotype show the detail of the boxed areas in the left panels. Granular nuage is indicated by white arrowheads; normal 6 dpf nuage is indicated by black arrows. Black arrowhead indicates a patch of darker nuage embedded within granular nuage (also see Fig. S2). Scale bars: 2 µm (overview); 1 µm (detailed images).

Tdrd1 is expressed only zygotically, starting around 3 dpf (Huang et al., 2011), coinciding with the emergence of compact nuage (Fig. 2B). In adults, Tdrd1 is found in the compact nuage that is typical for adult germ cells; in *tdrd1* mutant adult germ cells, no nuage can be detected (Huang et al., 2011). We tested the effect of Tdrd1 on the described nuage dynamics in PGCs using EM. As in the adult, *tdrd1* mutant PGCs at 6 dpf did not have detectable compact nuage (Fig. 3A,B, Fig. S2). However, these PGCs did contain the granular nuage that is typical of 1 dpf PGCs (Fig. 2A,B).

We also tested whether Zili, which (like Tdrd1) only starts to be expressed at 3 dpf (Houwing et al., 2008), is required for this shift in nuage morphology. Indeed, loss of Zili led to a very similar phenotype at 6 dpf, where only granular nuage and no compact nuage can be detected (Fig. 3C, Fig. S2). Interestingly, in some cells we did observe small patches of darker nuage embedded in granular nuage (Figs S2,3C), similar to the small patches of dense nuage we observed in wild-type PGCs at 3 dpf (Fig. 2F). In contrast, homozygous *ziwi* mutant PGCs did not show this phenotype, and contained normal adult-like nuage (Fig. 3C, Fig. S2). This lack of a phenotype likely stems from maternal Ziwi deposition (Houwing et al., 2007). Overall, these results show that the piRNA pathway is an important factor behind the observed changes in nuage morphology in germ cells, and that different Tudor-domain proteins mark different types of nuage.

### PGCs show increase in RNA Pol2 activity between 1 and 10 dpf

We observed a change in appearance of chromatin during PGC development in electron micrographs (Fig. S1D,E). This prompted us to investigate chromatin and RNA Polymerase 2 (Pol2) status in PGCs using immunohistochemistry. First, we analyzed H3K9 tri-methylation (H3K9me3) as a mark associated with heterochromatin and repressed transcription (Penagos-Puig and Furlan-Magaril, 2020). Using the signal from the somatic cells to normalize, we found that H3K9me3 in PGCs dropped between 1 dpf and 6 dpf (Fig. S3A,B). Additionally, H3K9me3 staining was diffuse in PGCs and only acquired the punctate appearance, which is visible in somatic nuclei, in some PGC nuclei at 10 dpf (Fig. S3A, arrowheads). Second, we analyzed H3K4me2 and H3K4me3, marks associated with transcription (Sims and Reinberg, 2006). These marks did not show strong global dynamics in the PGCs (Fig. S3C,D), although some PGC nuclei showed stronger H3K4me3 signal at 6 and 10 dpf (Fig. S3D). Finally, we addressed Pol2 activity by staining for the elongation-specific Ser2P modification (Fig. S4A). PGCs at 1 dpf had levels of Pol2Ser2P that are similar to the levels observed in their somatic neighbors. Starting in some cells at 3 dpf (Fig. S4A, right-most cell; Fig. S4B), and in all cells at 6 dpf, we observed increasing Pol2 elongation activity in PGC nuclei compared with that in the neighboring somatic nuclei. At 10 dpf, some cells still showed high Pol2Ser2P levels, whereas others returned to lower levels (Fig. S4A). Quantification of these experiments is difficult, because the somatic cells that we use for normalization may also display global chromatin changes. However, the relative loss of H3K9me3 in PGCs is consistent with the observed loss of electron-dense chromatin in the EM experiments, and the strong Pol2Ser2P signal starting at 3 dpf suggests that transcriptional strongly increases at that point in time. Therefore, we next aimed to analyze the transcriptional output of the PGCs at the different developmental stages.

### Germline genes are upregulated from 3 dpf onwards

To explore the transcriptional landscape of PGCs, we created small RNA-, total RNA- and polyA-selected RNA libraries, all originating from the same samples of FACS PGCs (Fig. S4A) and whole embryos at different time points in triplicate. We chose 0 hpf (1 cell stage) to obtain insight into what is maternally provided and 1, 2, 3, 6 and 10 dpf to explore the transcriptome at different developmental stages. First, we will describe gene expression as obtained from the polyA-selected libraries.

**Fig. 4.**
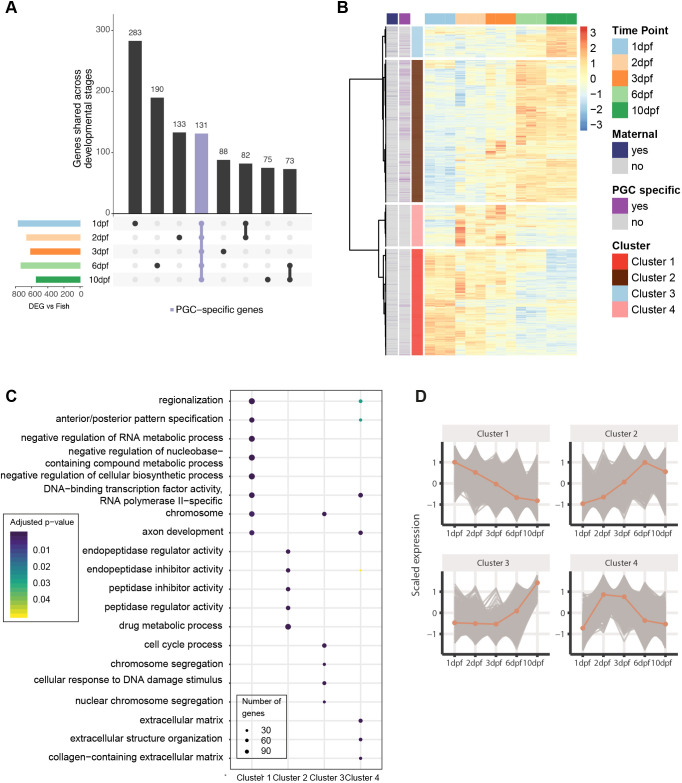
**Gene expression analysis in PGCs.** (A) UpSet plot of PGC-specific genes. The colored bar plot on the left represents the number of germline-enriched genes at indicated time points, determined by comparing with ‘whole fish’ at the same time point. The vertical bars represent the number of PGC-specific genes present at certain time points (dots) or at more than one time point (thick line). 131 genes are expressed at all five time points and enriched in the germline (purple; PGC-specific stable genes). (B) Hierarchical clustering of genes differentially expressed in the PGCs. Maternally provided annotation obtained from Aanes et al. (2011); PGC-specific genes, as defined by our own data, are indicated in the left two columns. (C) Gene ontology term enrichment of genes in the four different clusters. (D) Average scaled expression (*z*-score) of genes belonging to particular clusters.

To identify germline-specific genes, we compared gene expression between ‘PGC’ and ‘total fish’ samples at matched time points. Using a stringent cut-off of false discovery rate (FDR) of 0.01 and a fold change increase of more than 30, we found 1600 genes that were specifically expressed in PGCs throughout the time course. Almost half of these 1600 genes were specific for one developmental time point (769/1600 genes), but we also found 131 PGC-specific genes that were stably expressed in PGCs over the entire period. We called this set PGC-specific stable genes (PSGs) (Fig. 4A and Table S1). The PSGs include all known piRNA pathway members and many known germline genes, such as *zili*, *ziwi*, *tdrd1*, *tdrd9*, *vasa*, *tdrd6a*, *henmt1*, *rnf17*, *gtsf1*, *dnd1* and *dmrt1*. Additionally, a number of transcription factors, like *zglp1*, *zgc:171506* and the TATA box binding protein like 2 (*tbpl2*), are among these genes.

We then investigated how genes that were expressed in PGCs, but that were not necessarily PGC specific, behaved throughout the time course. To do this, we used hierarchical clustering to group genes into four clusters based on their expression patterns at the different time points (Fig. 4B and Table S2). Cluster 1 was composed of many genes whose transcripts are known to be maternally provided via the germ plasm, such as *granulito*, *nanos3*, *tdrd7a* and *ca15b* (Wang et al., 2013). As such, this cluster was characterized by genes whose expression declines during the developmental stages studied here.

Cluster 2 contained genes that are expressed at relatively low levels at 1 dpf, increasing in expression until they peak at 6 dpf. This cluster contained many of the known germline genes and factors involved in the piRNA pathway. Previous studies have shown that the expression of many piRNA pathway components start around 3 dpf (Houwing et al., 2008; Huang et al., 2011). This is also true in our datasets, but the effect at 3 dpf was overshadowed by an even stronger increase in expression at 6 dpf (Fig. 4D).

Cluster 3 genes remained low in the first few days, started to increase at around 6 dpf, and continued to rise until 10 dpf. These genes were enriched for GO-terms including cell cycle process, (nuclear) chromosome segregation and cellular response to DNA damage stimulus (Fig. 4C). These terms fitted well with the fact that, at this time, PGCs have been shown to become mitotically active again (Leerberg et al., 2017). Other noteworthy genes in this cluster were *ddx6* and *dcp1a*. Both proteins encoded by these genes are present in so-called piP-bodies in mouse. The piP-body in mouse not only contains the Piwi protein MIWI2, the tudor domain protein TDRD9 and MAEL, but also classical components of P-bodies, i.e. GW182, XRN1 and the above mentioned DDX6 and DCP1a (Aravin et al., 2009). We hypothesize that these observations are related to the changes in nuage that we detected in our EM experiments (Fig. 2). Cluster 3 also included *kdm4aa*, a member of the JmjC family of histone demethylases that, in other organisms, is involved in control of heterochromatin organization. KDM4A antagonizes H3K9 tri-methylation at pericentromeric heterochromatin in mammalian cells (Fodor et al., 2006; Klose et al., 2006). Expression of this gene could play a role in the observed loss of heterochromatin seen in EM (Fig. S1) and H3K9me3 stainings (Fig. S3A,B), although this idea needs experimental validation.

Cluster 4 contains genes whose expression peaked primarily at 2-3 dpf. GO term analysis revealed enrichment for genes involved in RNA polymerase II transcription factor activity and sequence-specific DNA binding (Fig. 4C). Interestingly, *prdm1a* and *prdm1b*, two homologs of the mouse transcriptional repressor *blimp1/prdm1*, which is required for germ cell development, were also part of this cluster. It is known that PGCs in zebrafish can lose their germ cell fate if they do not reach the genital ridge (Gross-Thebing et al., 2017). It is possible that PGCs only become fully committed germ cells once they interact with the cells at the genital ridge at this point in development and that the cluster 4 genes play a role in this process.

### Transcription of large intergenic regions starting at 3 dpf

**Fig. 5.**
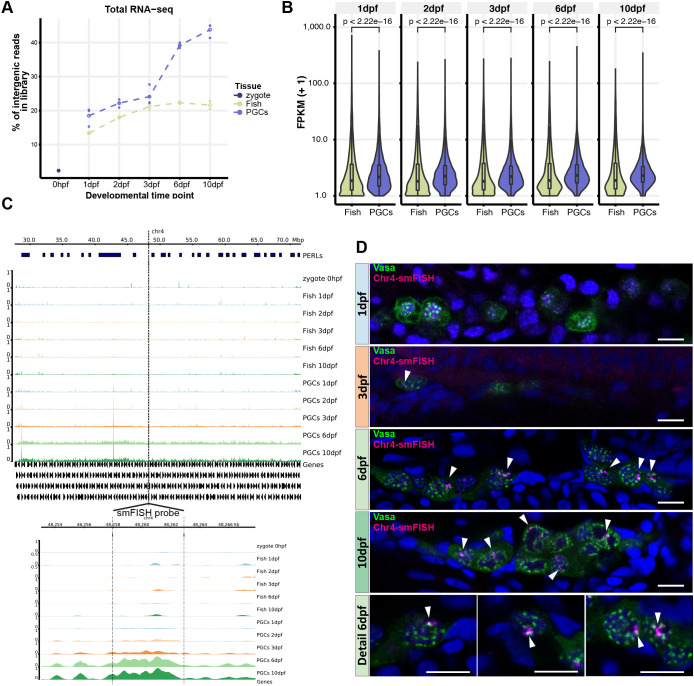
**Expression of intergenic regions in the PGCs.** (A) Proportion of intergenic reads in total RNAseq libraries at the different indicated time points. (B) Distributions of the expression levels of intergenic regions at the different time points in fragments (+1) per kilobase of transcript per million mapped reads (FPKM). Significance of the differences between ‘Fish’ and ‘PGC’ samples was tested using a Mann–Whitney-Wilcoxon test. (C) Examples of the right of chromosome 4 as a region with many PERLs. The region targeted for smFISH is indicated with a dashed line and enlarged in the bottom panel. It does not overlap with the PERLs, as annotated by our scripts, but still shows a clear increase in expression. (D) Immunostaining for Vasa, combined with smFISH using a probe designed to target transcripts derived from chr4: 48256970-48262902. White arrowheads indicate smFISH signal. Scale bars: 10 µm.

Next, we analyzed rRNA-depleted libraries (referred to as ‘total RNA’) from the same time points. As rRNA is very abundant, its depletion is needed to reliably detect non-poly-adenylated transcripts, e.g. intergenic transcripts. The zygote was almost entirely deprived of intergenic transcripts (Fig. 5A); at 1, 2 and 3 dpf, slight increases in intergenic transcription in both PGCs and ‘whole fish’ samples were detected. At 6 dpf, a striking increase in intergenic expression was observed only in the PGCs; this further increased at 10 dpf. This increase in intergenic transcription was not an artefact caused by the increase of only a few loci, which could represent non-annotated genes, as the number of intergenic loci in PGCs that followed this trend also increased over time (Fig. 5B, Fig. S5A). We conclude that intergenic transcription is strongly activated in PGCs, starting between 3 and 6 dpf.

Visual inspection on a chromosomal scale suggested that, starting at 3 dpf but more clearly visible at 6 dpf and 10 dpf, large intergenic regions were transcribed, specifically in the PGCs. This was readily visible, e.g. on the long arm of chromosome 4 (Fig. 5C), which is relatively gene poor and mostly transcriptionally silent (White et al., 2017), and is a major piRNA-producing region in adult testis and ovary (Houwing et al., 2007). However, on most other chromosomes, such regions could also be readily identified, such as on chromosome 18 (Fig. S5B) (also see next section). Interestingly, transcription from these regions came from both strands and, even though it is for a big part intergenic, it also covered genes (Fig. S5C-E). From here on, we will refer to this type of expression as non-genic transcription, to reflect that it is neither excluded from nor limited to genes. To independently confirm this non-genic expression, we designed smFISH probes against an expressed region on chromosome 4 (Fig. 5C) and used them to visualize expression of this locus throughout the time course. There was no detectable expression in either PGCs or somatic cells at 1 dpf. At 3 dpf we detected smFISH signal in a restricted number of PGC nuclei; at 6 and 10 dpf, all PGCs were positive for the smFISH signal (arrowheads in Fig. 5D). Consistent with our total RNAseq results, the smFISH signal was restricted to PGCs. At a subcellular level, the signal was found within the nucleoplasm, overlapping with DAPI, but also in perinuclear granules that were positive for Vasa (Fig. 5D). These findings are consistent with the possibility that these transcripts are exported to nuage, where they may be processed into piRNAs. To address this possibility, we aimed to systematically identify such transcribed non-genic loci and to relate them to piRNA expression.

### PERLs are transposon-rich piRNA-producing loci

**Fig. 6.**
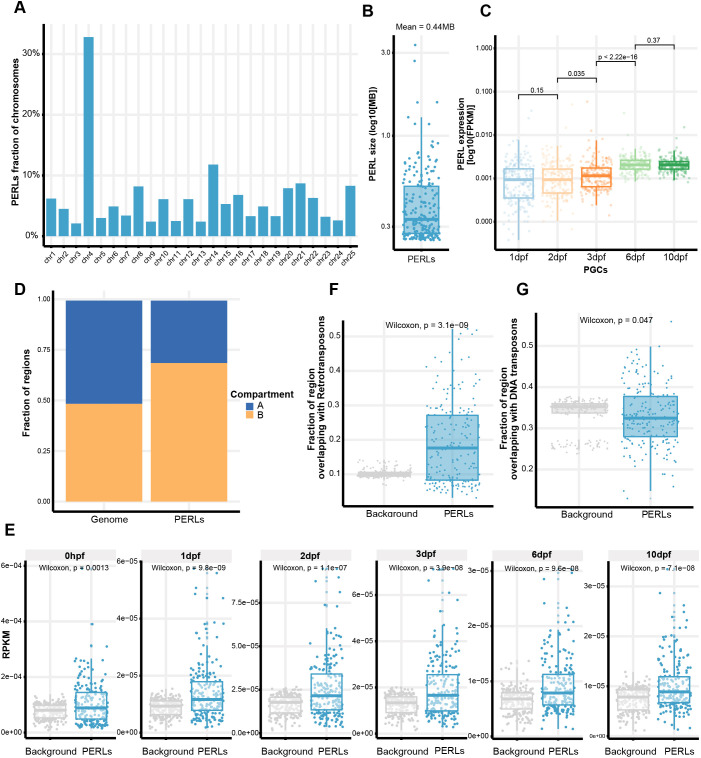
**PERLs are transposon-rich piRNA-producing loci.** (A) PERLs as a proportion of all zebrafish chromosomes. (B) Size distribution of PERLs. (C) Expression levels of PERLs across the different time points. Significance was tested with a Wilcoxon test. (D) Fraction of PERL base pairs that overlap with either A or B compartments. The number of genomic bases that belong to either of those compartments is also shown for comparison. (E) piRNA density (in RPKM) of PERLs compared with non-PERL loci, at the indicated time points. (F,G) Fraction of PERL bases that overlap with (F) RNA or (G) DNA transposons. Background is a set of size- and chromosome-matched random regions.

We next aimed to define loci that display dynamics as shown in Fig. 5A in a structured manner, using a set of pre-defined criteria. We first identified large (>250 kb) nearly contiguous regions of expression in both PGCs and ‘whole fish’ separately, and excluded those that are found in both these samples to isolate PGC-specific regions (for details see Materials and Methods). Next, regions defined at different time points were combined to generate one set of loci, that we from now on will refer to as PERLs (PGC-expressed non-coding RNA loci). Visual inspection revealed that most of these PERLs indeed show the required characteristics, but we also noticed our criteria may underestimate the size of PERLs, or fragment them (e.g. Fig. 5C). It follows that genuine PERLs may be missed by our analyses. Nevertheless, the set of PERLs that we define is a fair representation of the broad expression phenomenon we discovered in the PGCs. In total, we identified 201 PERLs (Table S3), deriving from all the zebrafish chromosomes (Fig. 6A). With the exception of chromosome 4, where PERLs occupy roughly one-third of the chromosome, in all other chromosomes the fraction of PERLs ranged between 2 and 12% (Fig. 6A). Their size ranged between our minimum cut-off of 250 kb and up to nearly 3 Mb (Fig. 6B), and their expression strongly increased at 6 dpf (Fig. 6C).

Given that heterochromatin visually disappears in the PGCs, and that heterochromatin has been proposed as a key driver in A/B compartmentalization (Falk et al., 2019), we tested whether PERLs may correlate with the A (generally open) or B (generally closed) status of chromatin, as defined from HiC analysis on embryonic samples (Kaaij et al., 2018). Although the zebrafish genome is evenly split between A/B compartments, PERLs were over-represented in B compartments (Fig. 6D).

Our smFISH experiments lead us to speculate that PERL transcripts could be exported to nuage where piRNA processing is known to occur (Ozata et al., 2019). To address the hypothesis that PERL transcripts are piRNA precursors, we compared piRNA coverage (also see next section) in PERLs with that of a set of randomly selected genomic regions. Indeed, there was significantly higher piRNA expression from PERLs than expected by chance, and this phenomenon was consistently found for piRNA populations from different stages of development (Fig. 6E). Interestingly, PERLs were also significantly enriched for retro-transposons, which are the major piRNA targets in zebrafish (Houwing et al., 2007; Kaaij et al., 2013), but not for DNA transposons (Fig. 6F,G). Given that DNA transposons cover a significantly larger fraction of the genome than retro-elements (Howe et al., 2013), this enrichment could indicate that retro-elements play a role in driving PERL expression. We conclude that PERL transcripts are excellent candidates for being the piRNA precursors in the zebrafish.

### Evidence for zygotic piRNA pathway activation at 6 dpf

**Fig. 7.**
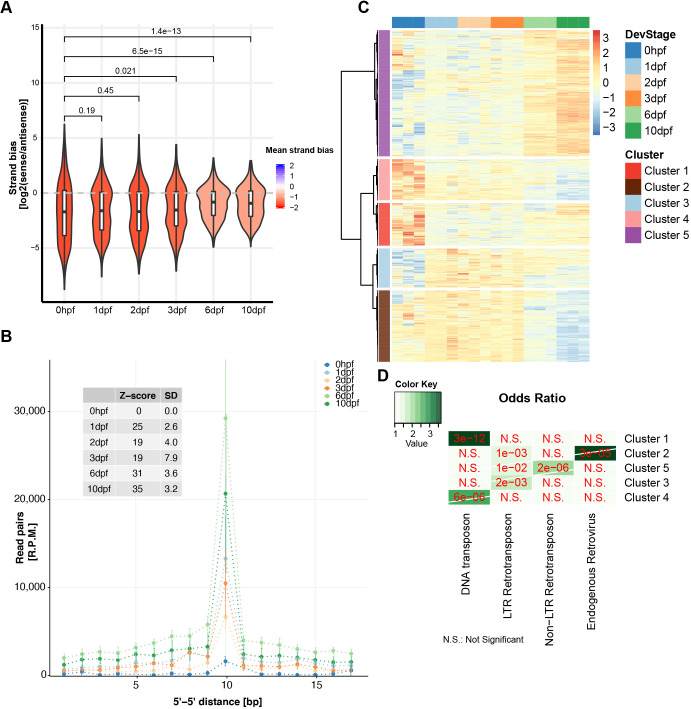
**Zygotic piRNA pathway activity.** (A) Sense/antisense bias of piRNAs at the indicated time points (*P*-values generated using the Wilcoxon test). (B) Ping-pong signature was assessed at the different time points by plotting how many pairs of piRNAs from opposite genomic strands (*y* axis) display the overlap of a certain number of bases at their 5′ ends (*x* axis). *Z*-scores were calculated to test significance. Data are mean±s.d. for three replicates. (C) Hierarchical clustering of TE using the number of piRNAs mapping to those elements at the different time points. (D) Enrichment analysis of TE in the piRNA target clusters in C.

Finally, we used small RNA-sequencing data to analyze piRNA dynamics in PGCs, from 1 to 10 dpf. Given the limited amounts of biological material, Ziwi and Zili immunoprecipitation was not possible. To circumvent this experimental limitation, and still enrich for bona-fide piRNAs over potential non-specific small RNA fragments, we concentrated on TE-derived small RNAs. These small RNAs displayed, at all time points, the characteristic Gaussian distribution, with a peak at 27 nucleotides (Fig. S6). From previously published work, we know that the piRNA pool that is maternally provided via Ziwi is heavily antisense biased (Houwing et al., 2007), and this bias we also observed at 1 and 3 dpf (Fig. 7A). Interestingly, at 6 dpf this antisense bias started to decrease significantly (Fig. 7A), accompanied by an increase in ‘ping-pong’ signature: the characteristic enrichment of a 10 bp overlap of the 5′ ends of piRNAs, caused by the nuclease properties of Piwi proteins (Fig. 7B). These results, combined with the already described increase in transcription of piRNA pathway components, are clear indications that, between 3 and 6 dpf, the piRNA pool starts to shift from purely maternally derived material to zygotically produced piRNAs.

We then investigated how the TE-derived piRNA repertoire changed throughout the time course. We grouped piRNAs into five different clusters using hierarchical clustering (Fig. 7C), and individual clusters were probed for enrichment of TE clades (Fig. 7D). Cluster 5, which was enriched for LTR- and non-LTR-retrotransposons (*P*<1e−02 and 2e−06, respectively, hypergeometric test, Fig. 7D) strongly gained piRNAs at 6 and 10 dpf (Fig. 7C), consistent with the enrichment of retrotransposon enrichment within PERLs (Fig. 6F). Indeed, these type of elements have been described before as major substrates in the zebrafish ping-pong cycle (Houwing et al., 2007, 2008). Interestingly, piRNAs from other TE clusters, including DNA transposons, endogenous retroviruses and also LTR retrotransposons, are enriched at earlier timepoints, pointing at potential differential activation of different transposons in the course of development. We conclude that between 3 and 6 dpf, the zygotic piRNA pathway is activated in zebrafish PGCs, and that PERLs are likely substrates in this process.

## DISCUSSION

After their arrival at the genital ridge, we found that zebrafish PGCs undergo many changes, both morphologically as well as molecularly. Our study reveals many interesting, thus far hidden, characteristics in zebrafish germ cell development, providing many openings for further studies on zebrafish PGC development. Some of these aspects will be further discussed below.

### piRNA pathway activation in zebrafish PGCs

We have previously described that piRNAs, in particular those that are Ziwi bound, are maternally provided and can influence piRNA characteristics trans-generationally (Houwing et al., 2007; Kaaij et al., 2013). However, owing to experimental difficulties in manipulating this maternal piRNA pool, the function of the maternal piRNAs in zebrafish is still unclear. Studies in *Drosophila* have revealed that maternal piRNAs are very important in establishing TE silencing (Brennecke et al., 2008; Marie et al., 2016), and this maternal effect is thought to act through the activation of piRNA clusters by nuclear Piwi protein activity (Akkouche et al., 2017; Le Thomas et al., 2014). We show that zebrafish most likely lacks a nuclear piRNA mechanism, and hence, the question arises of how maternal piRNAs can be involved in establishing the zygotic piRNA system in this animal. We hypothesize that maternal piRNAs in zebrafish do that by triggering the ping-pong amplification mechanism, as basically all required ping-pong-related proteins start to be expressed at the same point in time. Important substrates for this process could be the PERL-derived transcripts. Interestingly, *pld6* is activated at this same point in time, suggesting that the ping-pong mechanism may be tightly coupled to Pld6-driven piRNA production in zebrafish, as in *Drosophila* (Han et al., 2015; Mohn et al., 2015). Finally, we note that absence of a nuclear piRNA branch is not zebrafish specific. In many fish genomes only two Piwi proteins can be traced, suggesting that their piRNA systems may parallel that of zebrafish. Similarly, the cnidarian Hydra expresses only two Piwi proteins, both of which localize exclusively perinuclearly; in addition, in the silk moth *Bombyx mori*, a nuclear piRNA pathway seems to be absent (Kawaoka et al., 2008; Lim et al., 2014).

### What are PERLs and how are they activated?

We identify PERLs: large loci (up to several megabases in size) that are expressed from both strands in 6-10 dpf PGCs. What is their function and how are they activated? Clearly, both questions are closely connected, and we cannot give definite answers. However, we hypothesize that the ‘why’ of PERLs is closely related to piRNA biology: they are rich in TEs, the piRNA coverage of PERLs is higher than that of other genomic loci and we detected PERL transcripts close to Vasa-positive granules. Furthermore, PERL transcripts start to be expressed when the zygotic piRNA machinery also starts to be transcribed, and when nuage becomes more compact, a process driven by known piRNA pathway components such as Zili and Tdrd1. Even though these are mostly correlations, and we have not yet addressed causality, these features mark PERLs as excellent piRNA cluster candidates.

We hypothesize that the answer to the ‘how’ question of PERL activation may lie in a generally increased accessibility of transcription factors to the genome and also to heterochromatic regions. In flies, piRNA clusters are heterochromatic, yet they are transcribed by a specialized transcription machinery (ElMaghraby et al., 2019); in the fission yeast *Schizosaccharomyces pombe*, heterochromatin is also linked to transcription of small RNA precursors (Keller et al., 2012). By analogy, PERLs may represent such heterochromatic, yet transcribed, loci in the zebrafish. Indeed, we do find PERLs enriched in typically ‘closed’ regions of the genome (B compartments), but more detailed analysis of chromatin state will be required to dissect chromatin states in the PGCs at various developmental stages. Such analyses may also provide explanations for the apparent lack of H3K9me3 foci in PGCs until 10 dpf. However, we cannot rule out that one or a limited number of transcription factors is responsible for PERL transcription, as A-Myb has been identified to drive pachytene piRNA cluster transcription in mice (Li et al., 2013).

Are PERLs found in other organisms? In mice, large intergenic regions, named DADs, have been described to gain accessibility in gonocytes (Yamanaka et al., 2019). Even though links between the opening of the chromatin, increased intergenic transcription and piRNA production were not addressed, it is possible that zebrafish PERLs are mechanistically and/or functionally related to DADs in mice.

### Nuclear gyration during PGC development

We describe exceptional nuclear folding of nuclei in PGCs. To our knowledge, such nuclear shapes have not been described before in healthy, normally developing, diploid cells. Somewhat similar nuclear shapes have been described in nuclei from cells of progeria patients, and in zebrafish mutants defective in a protease that processes LaminA (Tonoyama et al., 2018), but even in these cases the gyration was not as extensive as seen in the normally developing PGCs that we studied. How and why do the nuclei of the developing zebrafish PGCs become so gyrated? As we often detect nuage structures within the folds it is possible that deformations are triggered by strong RNA export forces acting on nuclear pores, or associated structures. The extensive intergenic transcription of PERLs that we observed might provide such forces, as very long piRNA precursors could, on the one hand, still be bound to the locus or to other nuclear structures, while on the other hand becoming engaged by factors resident in nuage. Data supporting such as scenario have been described in *Drosophila* (Zhang et al., 2012).

The observed nuclear deformations in PGCs could also be linked to changes in genome ‘rigidity’, due to the loss of overall heterochromatin. Genomes are divided into compartments at different scales (Kim and Dekker, 2018), and phase separation-based mechanisms driven by such compartments have been implicated in nuclear organization (Gibson et al., 2019). Large-scale loss of compartmentalization, e.g. owing to the loss of heterochromatin (Falk et al., 2019), could produce different mechanical properties in the genome and possibly more ‘fluid-like’ nuclear contents. Combined with strong transcriptional activity and export, this could trigger the observed nuclear shapes. Testing such hypotheses will require the identification of factors that drive the loss of heterochromatin and probing whether changes in the chromatin state are linked to changes in material properties.

### Establishment of germ cell fate in the zebrafish

How or when is germ cell fate established in zebrafish? Even though it has been well established that the germ plasm is required to specify germ cells, it is yet unclear when the cells that inherit this germ plasm commit to germ cell development. Mis-localization of PGCs during their migration to the genital ridge can result in ectopic PGCs, and these cells have been shown to take on somatic cell fates (Gross-Thebing et al., 2017). This suggests that germ plasm inhibits somatic fates, rather than that it directly imposes germ cell fate. We describe activation of many ‘typical’ germ cell genes only at 3 dpf and not before, and that at this time also homologs of genes known to drive germ cell fate establishment in mouse are transiently expressed. Examples of these genes are zebrafish homologs of mouse *prdm1* and *14* (*prdm1a* and *b*, and *prdm8*) and *ap2γ* (*tfap2a-e*), that are known to be required for expression of *nanos3*, *dazl* and *ddx4*/*vasa*. Interestingly, at this point in time PGCs are extensively enclosed by somatic cells. Possibly, these somatic cells represent niches from which the PGCs receive signals that trigger their further development. The identification of molecular markers for these somatic cells will be required to help elucidate their role in germ cell development in zebrafish. We note that such relatively late establishment of germ cell fate may not be zebrafish specific, as also in mice, fixation of germ cell fate has recently been described to occur much later than was thus far assumed (Nicholls et al., 2019).

## MATERIALS AND METHODS

### Zebrafish lines

Zebrafish strains were housed at the Institute of Molecular Biology in Mainz and bred and maintained under standard conditions (26-28°C room and water temperature, and lighting conditions in cycles of 14:10 h light:dark) as described by Westerfield (1995). AB was used as wild type. Only fish less than 2 years old were used. Larvae <5 days post-fertilization were kept in E3 medium (5 mM NaCl, 0.17 mM KCl, 0.33 mM CaCl_2_ and 0.33 mM MgSO_4_) at 28°C. The *vasa:egfp* line was used for FACS (Krøvel and Olsen, 2002). All experiments were conducted according to the European animal welfare law, and were approved and licensed by the ministry of Rhineland-Palatinate. Mutant alleles were all previously published premature stop alleles: *ziwi(hu2479)* (Houwing et al., 2007), *zili(hu3173)* (Houwing et al., 2008) and *tdrd1(fh224)* (Huang et al., 2011). All animal experiments were performed in adherence to the animal welfare rules of Rheinland Palatinate.

### Immunostaining

Embryos were fixed in ice-cold 4% PFA in PBS (pH 7.4) or in 80% methanol/DMSO for 3 h on ice with gentle agitation, washed with PBST (PBS and 0.1% Tween20), dehydrated in a standard methanol series and stored for at least 12 h at −20°C. Whole-mount staining was performed using a modified protocol from Inoue and Wittbrodt (2011).

Embryos were rehydrated from methanol and washed with PBSTw twice for 10 min and for 2×10 min with PBS 1% Triton X-100. Embryos older than 24 hpf were decapitated and their tails were cut off for better penetrance of the solutions. Afterwards, antigen retrieval was performed with a 5 min incubation in 150 mM Tris-HCl (pH 9) at room temperature followed by 15 min at 70°C with gentle agitation. After washing with PBSTw, embryos were put in blocking solution (PBSTw with 1% BSA, 10% sheep serum, 0.8% Triton-X100) for at least 90 min followed by incubation with primary antibodies in blocking solution overnight at 4°C. After extensive washing with PBSTw (6×30 min), embryos were incubated with secondary antibodies overnight at 4°C. After another wash, DAPI was used to stain DNA. After additional washes, embryos were mounted ProLong Gold antifade mountant and imaged on a Leica SP5 confocal microscope with a 40× water objective (NA 1.3). Commercial primary antibodies that were used in this study (all used at 1:1000) were obtained from AbCam and GeneTex: anti-lamin B1 (ab16048; GR323340-1), anti H3K9me3 (ab8898; GR131339-1), anti-H3K4me3 (ab8580; GR188955-1), anti-H3K4me2 (ab32356[Y47]), anti-Vasa (GTX128306; 41290) and anti-RNA polymerase II CTD S2P (ab5095; GR231750-6). Secondary antibodies were obtained from Life Technologies [A21058(982289), A21094(1301836), A21434(Lot unknown) and A21428(1391902)] and Invitrogen [A11008 (564514)]. Previously published custom antibodies that were used: Tdrd6a-rabbit (Roovers et al., 2018) 1:400; Ziwi-rat-396 (Roovers et al., 2018) 1:250; Zili-rabbit-15 (Houwing et al., 2008) (1:500); and Tdrd1-rabbit (Huang et al., 2011) 1:100.

Immunostaining analysis was carried out as follows. Regions of interest (ROIs) were drawn for somatic cells and PGCs, and the area, mean, min and max intensity levels of the Pol2Ser2P channel were measured with ImageJ. The mean level of intensity between PGCs versus somatic cells was calculated and expressed as a ratio. Three measurements at each time point for somatic cells and PGCs were taken and ratios were compared between 1 and 6 dpf. For the three data points, Pol2Ser2P intensities from seven, 10 and nine PGCs (1 dpf), and nine, 11 and six PGCs (6 dpf) were compared with the intensities of somatic cells in the same image. Plotting and statistical testing was carried out in R using ggplot2 plugin (Wickham, 2016).

### Confocal imaging

Samples were imaged using a TCS SP5 Leica Confocal microscope with a 40× oil (NA of 1.3), 63× oil (NA of 1.4) or a 40× water (NA 1.2) objective. The following figures were deconvolved using Huygens software: Figs 1A,D, 3B, 5D.

### Electron microscopy

Zebrafish embryos at indicated time points were killed on ice and immediately fixed with half-strength Karnovsky fixative (pH 7.4) (Karnovsky, 1965) and post-fixed with 1% OsO4 in 0.1 M cacodylate buffer (pH 7.4), dehydrated in an acetone series and embedded in EPON. Semi-thin sections (1.5 µm) were cut on a Reichert Ultracut 2040 and a Butler diamond knife (Diatome) until the desired area in the embryo was reached. Ultra-thin sections (90 nm) were cut on a Reicher Ultracut E and collected on pioloform-coated copper slot grids, dried and stained with lead citrate under oxygen-free conditions for 2 min. Sections were examined with a Zeiss LIBRA 120 and 2048×2048 pixel; 16-bit images were acquired with a Albert Tröndle Restlichtverstärker Systeme (TRS) ccd camera. Image analysis was performed using ImageJ. Nuclei were manually selected and electron-dense and non-electron-dense regions within nuclei were identified using thresholding. Following thresholding, particles with a minimum size of 0.03 µm^2^ were automatically selected and counted. Nucleoli were removed and, for each image, the area of particles was expressed relative to the total nuclear area. Values were collected and stored in Excel, and the R ggplot2 plug-in was used to create plots. Statistical testing was also carried out in R.

### Single molecule fluorescent *in situ* hybridization

Single molecule fluorescent *in situ* hybridization probes were designed against an intergenic region on chromosome 4 (danRer10 Chr4: 48257970-48262902) with Stellaris probe designer with 48 probes targeting a region spanning 5933 bases. Fixed, dehydrated and then rehydrated embryos (tail and head was cut off from embryos) were incubated in hybridization buffer for 30 min at 30°C. Primary antibody and Stellaris probes (125 nM probe) were hybridized in hybridization buffer overnight at 30°C. After incubation, samples were washed with wash buffer at 30°C twice, and secondary antibody and DAPI were added during the second wash step (45 min). After another wash step, embryos were mounted in anti-bleach GLOX buffer [10 mM Tris (pH 7.5), 2×SSC, 0.4% glucose] containing 1 μl glucose oxidase (stock 3.7 mg/ml), 1 μl of catalase suspension and 1 μl of Trolox (stock 200 mM in ethanol) per 100 μl of buffer.

### Fluorescence-activated cell sorting

The *vasa:eGFP* line (Krøvel and Olsen, 2002) was used for FACS of PGCs at different time points. Embryos were collected and incubated with TrypLE Express for 40 to 70 min, killed on ice and gently pipetted up and down with a glass pipet and/or a 200 µl low-retention pipet tip. After visual inspection, cell suspension was separated from trunks using a 100 µm sieve. Following another 5-15 min of digestion, FCS was added to 10% of total volume. Cells were spun down at 500 ***g*** for 5 min at room temperature, washed with PBS, resuspended in PBS with 2% FCS, put on ice and immediately subjected to FACS using a 85 µm nozzle on a BD FACSAria III SORP (Becton Dickinson). Between 1000 and 2500 cells were sorted directly into Trizol. RNA from sorted PGCs and whole embryos was extracted with Trizol and stored in MQ at −80°C until library preparation was carried out.

### NGS library preparation

#### Poly-A RNA-sequencing

NGS library prep was performed with SmartSeq2 RNA-Seq System following NuGen's standard protocol (M01406v2). Libraries were prepared with a starting amount of 1 ng and amplified in 12 PCR cycles. Libraries were profiled in a High Sensitivity DNA on a 2100 Bioanalyzer (Agilent Technologies) and quantified using the Qubit dsDNA HS Assay Kit, in a Qubit 2.0 Fluorometer (Life Technologies). Samples were pooled in equimolar ratio and sequenced PE for 2×75 cycles plus 16 cycles for the index read.

#### rRNA-depleted RNA-sequencing

NGS library prep was performed with NuGen Ovation SoLo RNA-Seq System following NuGen's standard protocol (M01406v2). Libraries were prepared with a starting amount of 1 ng and amplified in 14 PCR cycles.

Libraries were profiled in a High Sensitivity DNA on a 2100 Bioanalyzer (Agilent Technologies) and quantified using the Qubit dsDNA HS Assay Kit, in a Qubit 2.0 Fluorometer (Life Technologies). Samples were pooled in equimolar ratio and sequenced PE for 2×75 cycles plus 16 cycles for the index read.

#### Small RNA-sequencing

NGS library prep was performed with NEXTflex Small RNA-Seq Kit V3 following Step A to Step G of Bioo Scientific's standard protocol (V16.06). Libraries were prepared with a starting amount of 1 ng and amplified in 25 PCR cycles. Amplified libraries were purified by running an 8% TBE gel and size-selected for 18-40 nt. Libraries were profiled in a High Sensitivity DNA on a 2100 Bioanalyzer (Agilent Technologies) and quantified using the Qubit dsDNA HS Assay Kit, in a Qubit 2.0 Fluorometer (Life Technologies). Samples from 1, 3 and 6 dpf and samples for zygote and 10 dpf PGCs were pooled in equimolar ratio and sequenced on 2 NextSeq 500 Flowcell, PE for 2×75 cycles plus 16 cycles for the index read.

### Data processing and analysis

#### Small RNA-sequencing

Raw reads were checked for quality with FastQC before adapter trimming with cutadapt (v1.9.1) -a AGATCGGAAGAGCACACGTCT -O 8 -m 21 -M 51, followed by removal of sequences with low-quality calls using fastq_quality_filter (-q 20 -p 100 -Q 33) from the FASTX-Toolkit (v0.0.14). PCR duplicates were removed making use of UMIs added during library preparation by collapsing reads with the same sequence, including UMIs, using a combination of unix command-line programs. Processed reads were mapped to the Zv10 Danio rerio genome assembly with bowtie (v0.12.8) -q --sam --phred33-quals --tryhard --best --strata --chunkmbs 256 -v 2 -M 1.

To match each mapped read, i.e. small RNA, with a genomic element, we downloaded repeat element annotations the UCSC genome browser track RepeatMasker (Zv10) and gene annotations from iGenomes (Danio rerio, GRCz10). We then intersected mapped reads with these annotations with bedtools intersect -wa -wb -bed -f 1.0 with either the flags -s or -S to determine which small RNAs map sense or antisense, respectively, the annotated features. Length profiles were obtained by filtering reads mapping sense or antisense to genetic elements annotated as belonging to a particular transposon class/biotype, and summarizing their length.

Ping-pong signal was determined by calculating the base pair distance between the 5′ of piRNAs overlapping in opposite strands (Brennecke et al., 2007). The Z-score was calculated as described by Wasik et al. (2015), and is defined as Z=(P10-M)/SD, where P10 is the number of reads pairs with 10 nucleotides overlap, and M and S are the mean and standard deviation, respectively, of the number of read pairs with 1-9 and 11-30 overlap. Sense/antisense bias was determined by calculating the ratio of reads mapping in the same or the opposite strand for each annotated transposable elements (DNA or RNA in repeatmasker). To generated scatterplots comparing piRNA abundances in two conditions, small RNAs that mapped to transposable elements were quantified per element and normalized to mapped reads.

Nucleotide bias of piRNAs was determined by summarizing the number of times a base is present in any given piRNA position. For the downstream U bias, the downstream annotated genomic bases, in the same mapping strand of the piRNA, were used (Han et al., 2015; Mohn et al., 2015).

TE targeting was determined by estimating the number of piRNAs reads mapping to repeat elements using repEnrich v1.2 with default settings (Criscione et al., 2014). repEnrich uses a two-step approach mapping to the genome and to transposable element sequences, to use both uniquely mapping and multi-mapping reads, and accurately infer read counts on repetitive elements. Targeted TEs were clustered using hierarchical clustering in R [hclust(as.dist(1-cor(t(rldLRT_05), method=“pearson”)), method=“ward.D”)].

#### mRNA-seq and total RNA

mRNA and total RNA read processing and mapping library quality was assessed with FastQC before being aligned against the *D. rerio* genome assembly and the genome annotation (Zv10/GRCz10) with STAR v2.5.2b (–runMode alignReads –outStd SAM –outSAMattributes Standard –outSAMunmapped Within –outSJfilterReads Unique –outFilterMismatchNoverLmax 0.04 –clip3pAdapterSeq CTGTCTCTTATACACATCT –sjdbGTFfile –sjdbOverhang 82). Read alignment was performed using the full genomes and reads mapping to unassembled contigs were removed after read alignment. mRNA Coverage tracks were generated with deepTools v2.4.3 (bamCoverage –smoothLength 60 –binSize 20 –normalizeUsingRPKM) (Ramírez et al., 2016), and total RNA coverage tracks were created with bamCoverage --binSize 50 --smooth Length 500 --normalizeUsing CPM --blackListFileName blacklisted.bed –effectiveGenomeSize 1369631918. blacklisted.bed is available at GitHub (https://doi.org/10.5281/zenodo.4395174). Stranded coverage tracks were created as above but deepTools's code was modified to allow for normalized of stranded signal to total mapped reads. Public datasets used for ovary and testis sashimi plots were obtained from SRR1524248 and SRR1524249 at the Sequence Read Archive at NCBI, and processed as described above.

#### Gene and transposon expression

Differential expression and clustering gene/transcript expression was quantified with salmon v0.11.0 (Patro et al., 2017) (salmon quant -l A -1) or salmonTE v0.2 (Jeong et al., 2017) for the transposable element expression (SalmonTE.py quant –reference=dr –num_threads=4 – exprtype=count). Differential expression analysis was performed with the Bioconductor package DESeq2 v.1.18.1 (love_moderated_2014). The following settings were used to determine differentially expressed genes, depending on the goal: (1) to determine which genes are changed during PGC development at any time point, we defined a reduced model in combination with a likelihood ratio test [DESeq(dds, test=“LRT”, reduced=∼1)]; (2) to determine which genes are PGC enriched, a simple pairwise comparison fish versus PGCs at matched time points was executed. Genes were clustered using hierarchical clustering in R [hclust(as.dist(1-cor(t(rldLRT_05), method=“pearson”)), method=“ward.D”)]. The list of maternally contributed genes was obtained from Aanes et al. (2011). To generate a list of genes that are very likely PGC specific, only genes with fc≥30 enriched in the PGCs (versus fish) and with fdr<0.01 were selected. These cut-offs were based on the most stringent threshold that would still be able to retain well-know PGC-specific genes. Normalized counts were obtained from DESeq2. Go term enrichment analysis for categories was performed with the Bioconductor package ClusterProfiler (Yu et al., 2012), using all genes with at least one count as the background set.

TE expression was quantified with salmonTE v0.11.0, SalmonTE.py quant --reference=dr --exprtype=count, which uses salmon to quantify TE and summarize according to TE element (Jeong et al., 2017). Hierarchical clustering was performed as described above for gene expression.

#### Intergenic expression

Intergenic regions were defined by extending any annotated region in the GRCz10 ensembl GTF by 2500 bp and then taking the complementary genomic locations (bedtools slop -i stdin -g chrom.sizes -b 2500|bedtools merge|bedtools complement). Non-annotated rRNA locations identified with blast (Table S4) and other contaminants were also discarded. Reads mapping to intergenic regions were counted with bedtools coverage -split -counts -F 1.0.

#### PERL identification

We identified the PERLs using a stepwise approach guided by our observations of large contiguous genomic region being expressed at certain time points, which included both genic and non-genic regions. (1) We used derfinder to call expressed regions separately for each time point and tissue (average coverage of the replicates). Derfinder calculates the mean (normalized) coverage at every genomic base. Bases passing the cutoff (0.25) and within 3000 bp of each other are then joined in a single region. (2) ERs that are close to each other by less than 3 kb were merged in a single contiguous expression cluster. (3) To identify only those regions specific to PGCs, at each time point the PGC clusters were overlapped with those of the Fish, and those that overlap by more than 10% of (PGC cluster) bases were excluded. (4) Clusters smaller than 250 kb are removed to keep only large PGC-specific expressed regions or PERLs. Finally, as the observed global expression tends to take place at later developmental stages (3-6), we excluded those regions also present at 1-2 dpf.

#### PERL characterization

Unless stated otherwise, as a background for comparisons, we created 100 sets of background regions matched for size and chromosome with the PERL regions. These background regions were generated with bedtools shuffle -i perls.bed -g -excl perls.bed -chrom. To estimate PERL coverage/expression, reads mapping to each PERL were counted with bedtools coverage -split -counts -F 1.0, normalized to size and library depth (RPKM), and the expression for each replicate was averaged. piRNA abundance in PERLs was determined using 23-33 small RNA reads with multiBigwigSummary BED-file. Coverage was then normalized for size and the biological replicates averaged, or, in the case of the background regions, the matched PERL regions were averaged. Statistical comparisons were made with the Wilcoxon rank test. The base overlap with transposons and A/B compartments was determined with coverageBed (fraction of covered bases).

## Supplementary Material

Supplementary information

Reviewer comments
