# Supplementary material for: Extensive nuclear gyration and pervasive non-genic transcription during primordial germ cell development in zebrafish
Source: Development. 2021 Jan 20;148(2):dev193060. doi: 10.1242/dev.193060 (PMC7847270; doi:10.1242/dev.193060)
Supplement: Supplementary information [file develop-148-193060-s1.pdf]

**Figure S1**

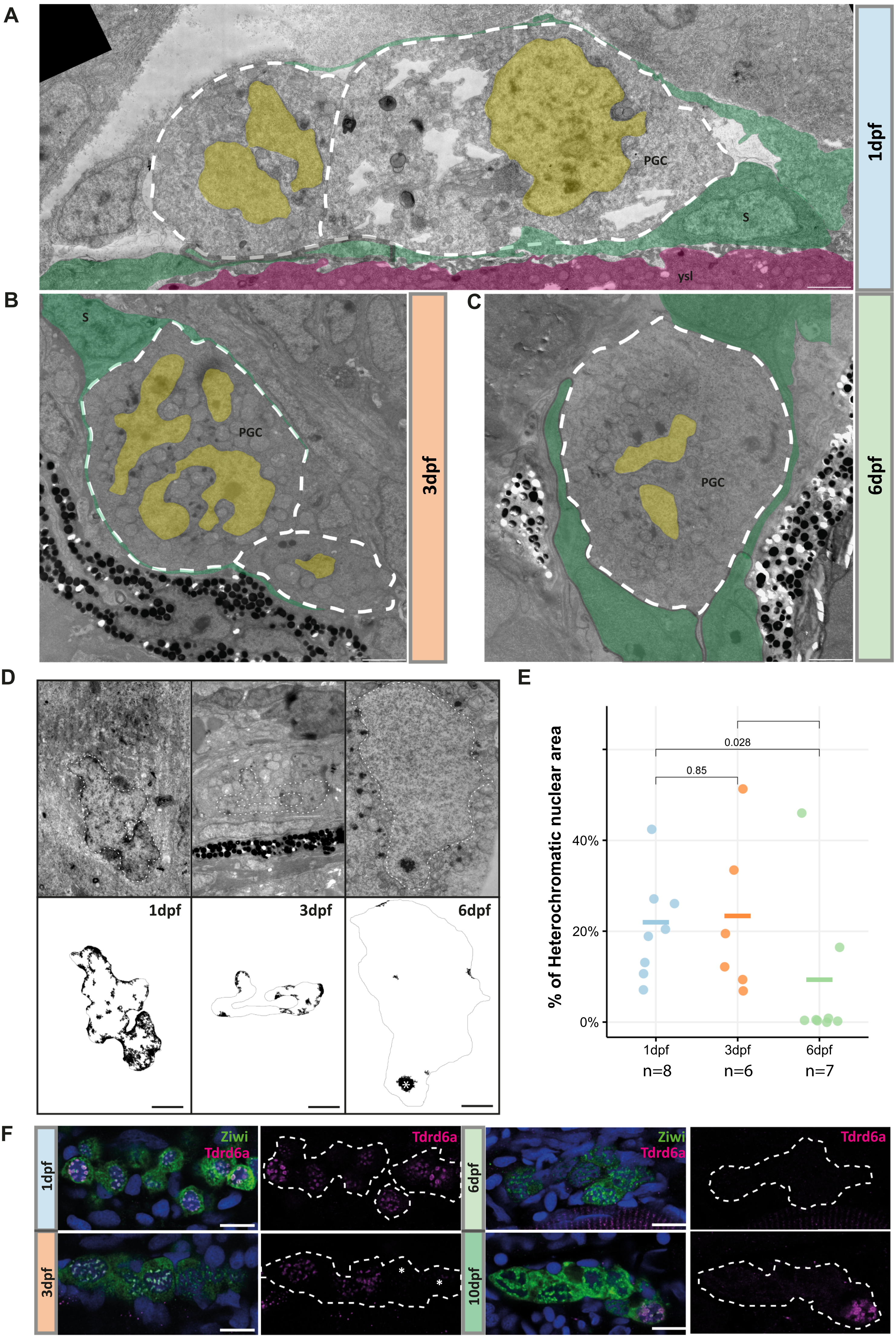


Development • Supplementary information

**Fig. S1: Electron micrographs of wild-type PGCs**

(A, B, C) Electron micrographs of PGCs at 1, 3 and 6dpf respectively. Yellow overlays mark nuclei, green overlays mark somatic cells that are in close association with the PGCs, magenta overlay marks yolk syncytial layer (ysl). (D) representative images of thresholding for heterochromatin analysis in PGCs. * marks a nucleolus, which was excluded from heterochromatin analysis and nuclear area. (E) Dot plot showing the percentage of heterochromatin of the total nuclear area in PGCs at indicated timepoints. Statistical significance of the differences between the indicated time points were calculated with the Mann-Whitney-Wilcoxon Test. (F) Double immune stainings in 1, 3, 6 and 10dpf PGCs for Ziwi (green) and Tdrd6a (magenta). Scale bars EM images: 2$\mu$m. Scale bars immune stainings: 10$\mu$m.

# Figure S2

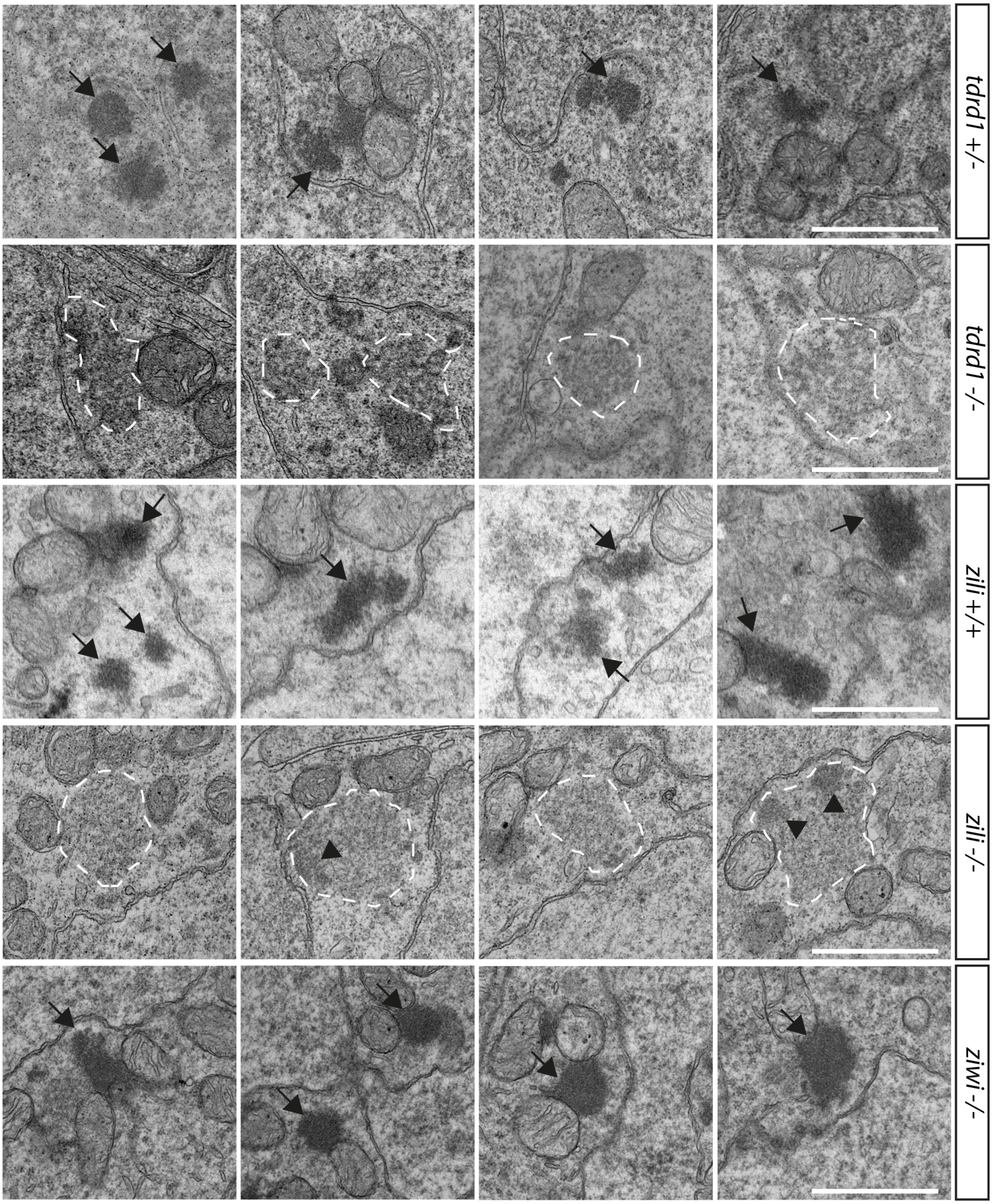

**Fig. S2: Electron micrographs of mutants PGCs**

Four examples of PGCs at 6dpf from the indicated genetic backgrounds, obtained from incrosses of heterozygous parents. +: wild-type allele; -: premature stop allele. Granular nuage, as normally seen up to 3dpf but not later, is encircled by a white dashed line; normal 6dpf-like nuage is indicated by black arrows. Black arrow heads indicate patches of darker nuage within granular nuage, as also seen in wild-type nuage at 3dpf (Fig. 2F). Scale bars 1$\mu$m. We note that the second image of the *zili* mutant contains a horizontal break in continuity approximately at the middle of the image. This is the result of image acquisition of larger images, where software needs to stich multiple images together. This process is sometimes not perfect and can create artefacts at the overlap.

## Figure S3

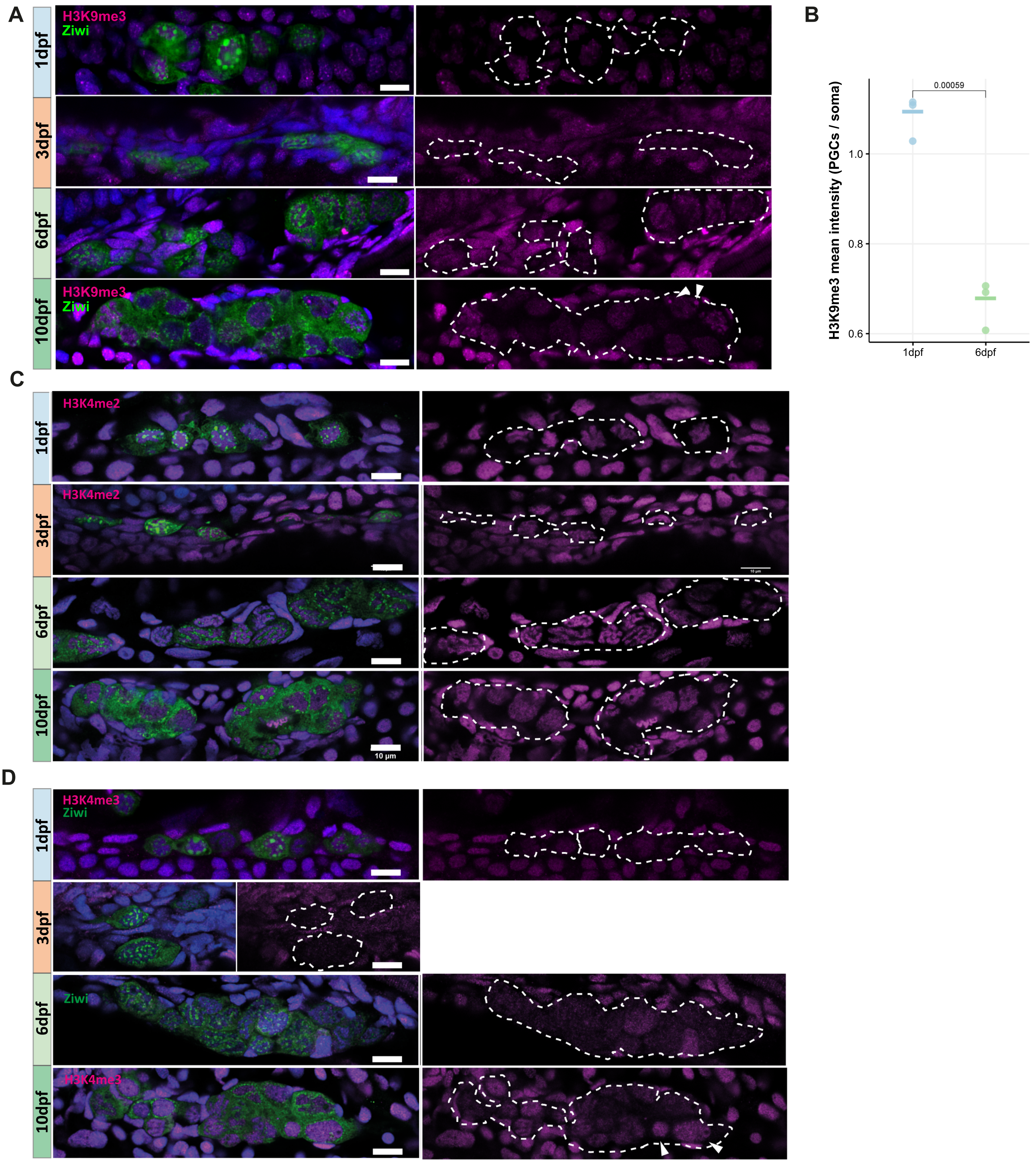
A
B
C
D
H3K9me3
Ziwi
1dpf
3dpf
6dpf
10dpf
H3K9me3 mean intensity (PGCs / soma)
0.00059
1.0
0.8
0.6
H3K4me2
10 µm
H3K4me3

**Fig. S3: Histone modifications in PGCs**

(A) Double immunostainings for H3K9me3 (magenta) and Ziwi (green) at indicated time points. Left panels: merge of all three indicated channels. Right panel: single channels of H3K9me3 staining. Blue: DAPI. The two arrow heads indicate two clearly distinct H3K9me3 foci. Scale bar: 10$\mu$m. (B) Intensity ratios of H3K9me3 stainings between PGCs and somatic cells at 1 and 6dpf. Data points represent PGC/soma ratios from 3 images with a total of 53 PGCs at 1dpf and 22 PGCs at 6dpf. (C) Double immune stainings for Histone modification H3K4me2 (magenta) and Ziwi (green) in PGCs at indicated timepoints. (D) H3K4me3 (magenta), Ziwi (green) double immune staining at indicated timepoints. Blue: DAPI. Scale Bar 10$\mu$m.

Development • Supplementary information

## Figure S4

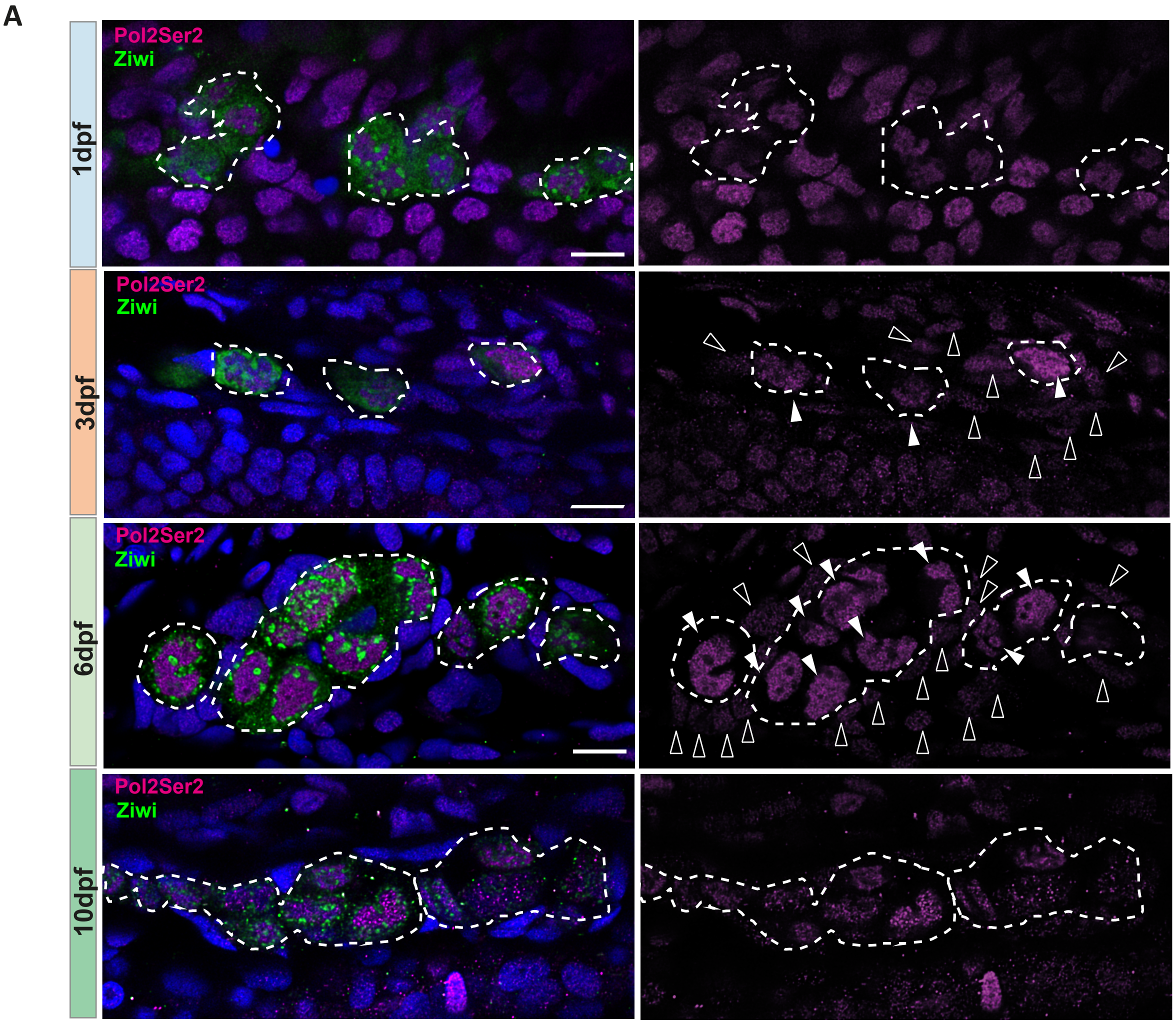


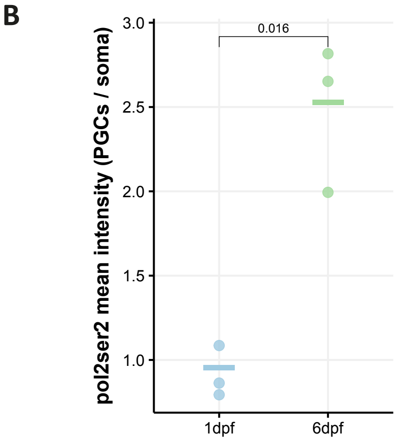


Development • Supplementary information

**Fig. S4: RNA Pol2 activity in PGCs**

(A) Immune stainings for Ziwi (green) and Ser2-P modification in CTD of RNA polymerase II (Pol2Ser2P) in PGCs at indicated time points. Left panel: merge of all three indicated channels. Right panel: single channels of Pol2Ser2 staining, where open arrow heads mark somatic nuclei and closed arrow heads PGC nuclei. Blue: DAPI. Scale bar: 10$\mu$m. (B) Intensity ratios of Pol2Ser2 stainings between PGCs and somatic cells at 1 and 6dpf. Data points represent PGC/soma ratios from 3 images with a total of 34 PGCs at 1dpf and 40 PGCs at 6dpf. The p-values in C and D were calculated with a two-sides t-test.

Development • Supplementary information

# Figure S5

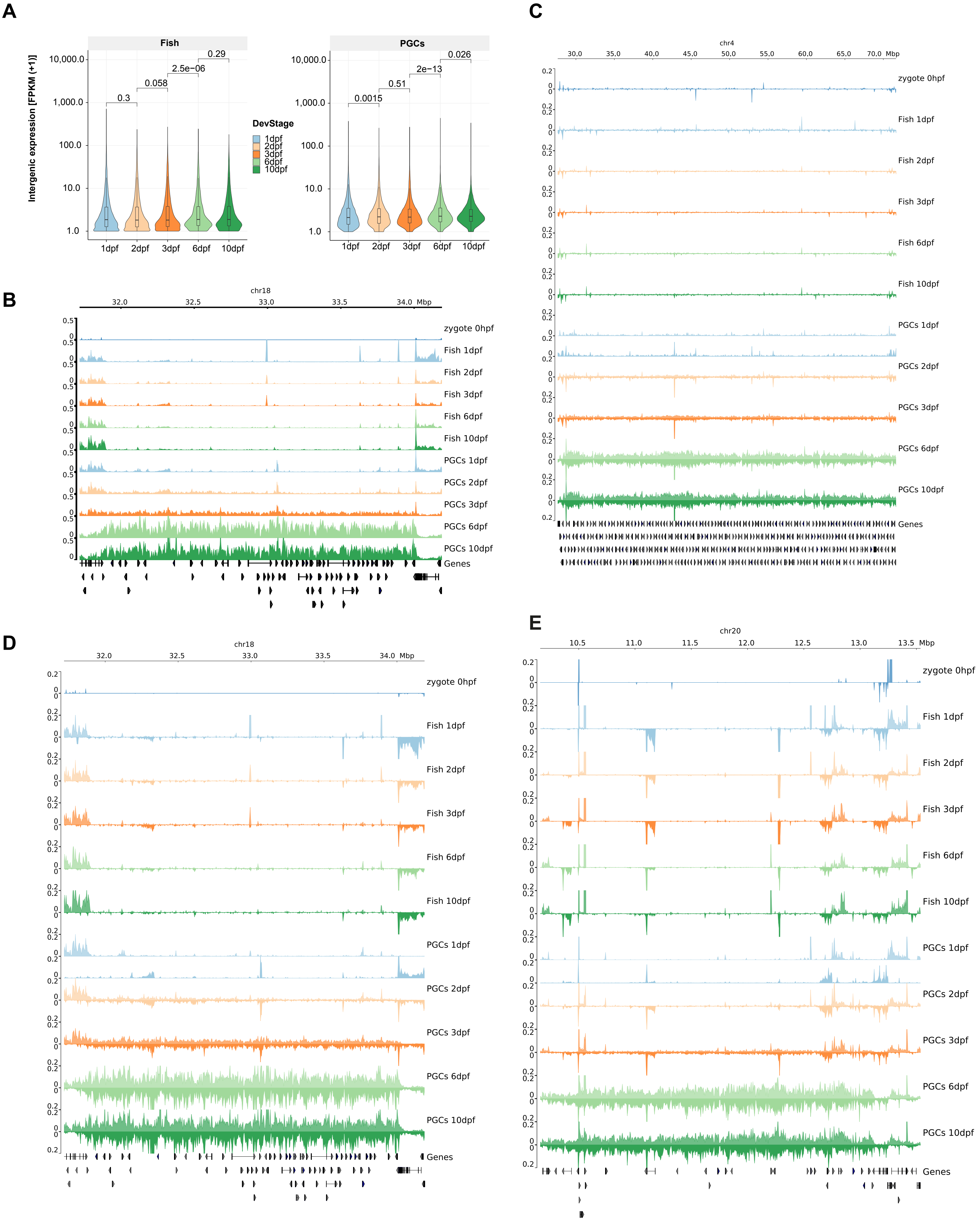


Development • Supplementary information

**Fig. S5: PERLs are transcribed from both strands**

(A) The same data as displayed as in Fig. 5B, but here the distributions between the different time points is compared, and tested using a Mann-Whitney-Wilcoxon test. (B) Coverage tracks showing a part of chromosome 18 and total RNA coverage at the different timepoints. (C) Stranded coverage tracks of the same region of chromosome 4 shown in Figure 5C. (D) Stranded coverage tracks of the same region of chromosome 18 shown in Figure S5B. (E) Stranded coverage tracks of right arm f chromosome 20.

Development • Supplementary information

## Figure S6

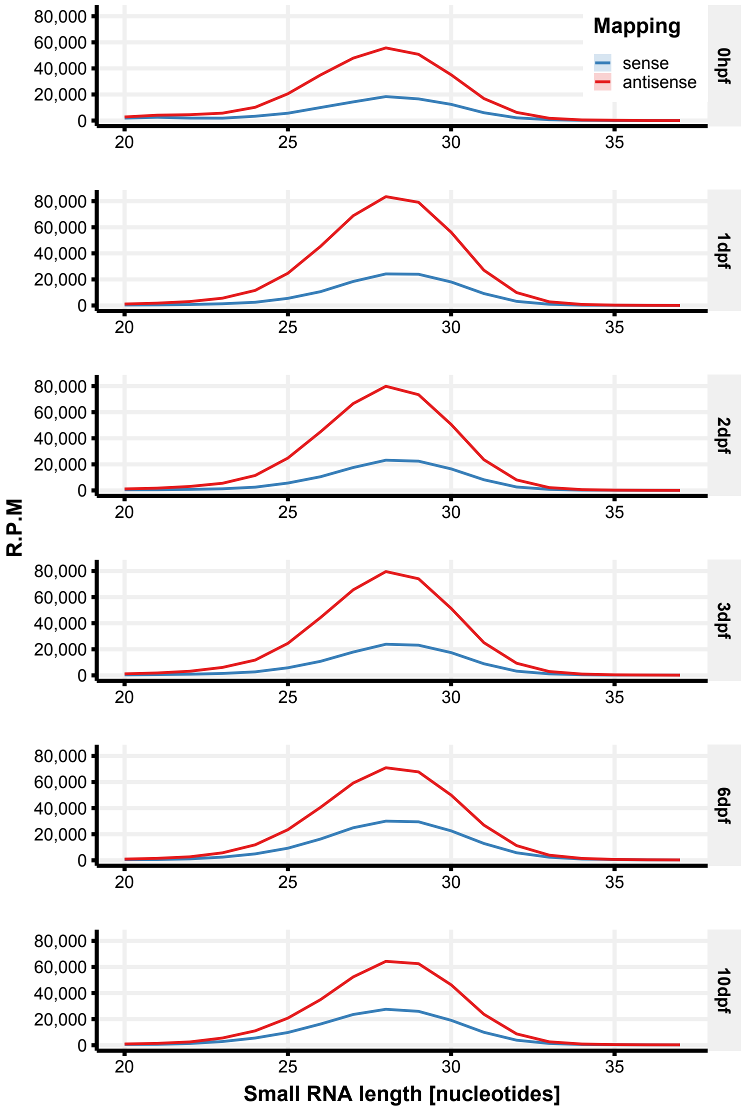


**Fig. S6: piRNA length profiles throughout the time course**

Length distribution of small RNAs reads that map sense (blue line) or anti-sense (red line) to TEs per time point in PGCs. Lighter shading indicates the standard deviation of three biological replicates.

Development • Supplementary information

**Table S1: PSG genes**

This tab-delimited file contains all the genes we marked as stably and specifically expressed in PGCs.

Click here to Download Table S1

**Table S2: IDs of genes linked to the four clusters we defined**

This tab-delimited file provides the information on which genes are in which cluster.

Click here to Download Table S2

**Table S3: PERLs**

This bed file contains the annotation for all the PERLs we identified.

Click here to Download Table S3

**Table S4: Non-annotated rRNA loci**

This bed file contains the annotations of non-annotated rRNA loci (see Methods).

Click here to Download Table S4
