# Supplementary material for: Extensive nuclear gyration and pervasive non-genic transcription during primordial germ cell development in zebrafish
Source: Development. 2021 Jan 20;148(2):dev193060. doi: 10.1242/dev.193060 (PMC7847270; doi:10.1242/dev.193060)
Supplement: Reviewer comments [file develop-148-193060_review_history.pdf]

Extensive nuclear gyration and pervasive non-genic transcription during primordial germ cell development in zebrafish

Stefan Redl, Antonio M. de Jesus Domingues, Edoardo Caspani, Stefanie Möckel, Willi Salvenmoser, Maria Mendez-Lago and René F. Ketting
DOI: 10.1242/dev.193060

Editor: Swathi Arur

Review timeline

Original submission:	19 May 2020
Editorial decision:	22 June 2020
First revision received:	2 November 2020
Editorial decision:	23 November 2020
Second revision received:	30 November 2020
Accepted:	2 December 2020

Original submission

First decision letter

MS ID#: DEVELOP/2020/193060

MS TITLE: Extensive nuclear gyration and pervasive non-genic transcription during primordial germ cell development in zebrafish

AUTHORS: Stefan Redl, Antonio Miguel de Jesus Domingues, Stefanie Moeckel, Willi Salvenmoser, Maria Mendez-Lago, and Rene Ketting

I have now received reviews of your manuscript from 2 experts. The reviewers' comments are appended below, or you can access them online: please go to BenchPress and click on the 'Manuscripts with Decisions' queue in the Author Area.

As you will see, both reviewers express interest in your study but have some concerns that you should address before I can consider it for publication in Development. Both note that your paper is full of valuable data but in its current form is mainly descriptive. Reviewer 1 suggests that one remedy would be to better connect the different findings in the paper to make the advance to the field more obvious. Reviewer 2 suggests that you move beyond description by including some experiments, analysis of at least one mutant, and/or functional analysis of an RNA or protein identified as potentially important in this work. In addition, both reviewers have questions about your experiments, findings, and figures, and make numerous excellent suggestions for improving your study and your manuscript.

I invite you to consider the reviewers' suggestions and submit a revised manuscript that addresses their concerns. Your revised manuscript would be re-reviewed, and acceptance would depend on your satisfactorily addressing the reviewers' concerns. Please note that Development normally permits only one round of “major revision”.

In your revised manuscript, please clearly HIGHLIGHT all changes made in the revised version. You should avoid using 'Tracked Changes' in Word files as these are lost in PDF conversion. I also request a point-by-point response detailing how you have dealt with the points raised by the reviewers in

© 2020. Published by The Company of Biologists under the terms of the Creative Commons Attribution License (https://creativecommons.org/licenses/by/4.0/).

the 'Response to Reviewers' box. If you do not agree with any of the reviewers' criticisms or suggestions, please explain why.

We are aware that you may currently be unable to access the lab to undertake experimental revisions. If it would be helpful, we encourage you to contact us to discuss your revision in greater detail. Please send us a point-by-point response indicating where you are able to address concerns raised (either experimentally or by changes to the text) and where you will not be able to do so within the normal time frame of a revision. We will then provide further guidance. Please also note that we are happy to extend revision time frames as necessary.

Reviewer 1

Advance summary and potential significance to field

In “Extensive nuclear gyration and pervasive non-genic transcription during primordial germ cell development in zebrafish”, the authors study germ cells from after their arrival at the genital ridge, until the time they start to form a definite gonad. In contrast to the initial specification and migration of PGCs in zebrafish that have been relatively well-studied, little is known about these later stages.

The manuscript contains a wealth of data including the discovery of extensive nuclear gyration in PGCs, changes in nuage morphology, the dynamics of gene expression in PGCs, and the identification of PERLs. These data will be important to further our understanding of germ cell development should be published without a doubt. We do feel, however, that the authors could try to connect the different findings in the paper better (mostly in the text) in order to better explain the advance it provides for the field.

Comments for the author

Below, we have listed some minor concerns / suggestions:

Figure 3 (and Figure S1)
Heterochromatin is analyzed by EM (Figure S1D,E) and IF (Figure 3A). The data of these two methods does not result in the same conclusion. A related issue concerns the analysis of the IF data: signal intensity in the PGCs is normalized to signal intensity in the soma. To justify this, the authors should show that the signal in the soma does not change over time. Visual inspection of the images in Figure 3A, however, suggests that this is not the case. Finally, we note that the almost unchanged H3K9me3 signal in PGCs observed does not support claims later in the manuscript that the increase in expression of the kdm4aa demethylase is driving the loss of heterochromatin. The authors should address these issues.

Figure 3A, legend
Please indicate what panels on the right are, like was done in 3B

Figure 3B, legend
Pol2Ser should be replaced with Pol2Ser2P to indicate the phosphorylation state which is the hallmark of transcription elongation.

Figure 3C
The quantification of signal is at odds with the emphasis on the K9me3 foci. Can the autors explain how they interpret both types of signal?

Figure S1D
It would be helpful to outline the nuclei that were analyzed.

Figure S1E
How many nuclei were analyzed?

Figure S2

© 2020. Published by The Company of Biologists under the terms of the Creative Commons Attribution License (https://creativecommons.org/licenses/by/4.0/).

The rationale for the H3K4me2 and H3K4me3 staining and the conclusions drawn from it could be better explained in the text. Is this the 'opening of chromatin' the authors write about in the abstract? Was the specificity of the H3K4me2 Ab tested?

Figure 4A, B
The color codes used for timepoints in panels A and B is different which is confusing.

Figure 4B and related text
Inconsistent use of cluster/group in the text (cluster 1, cluster 2, group 3, group 4).

Figure 5 and related text
The authors write “The zygote was almost entirely deprived of intergenic transcripts, as expected by the lack of zygotic transcription”. These reviewers does not understand this rationale. Could intergenic transcripts not be maternally loaded?

Figure 5A
This is not total RNA but rRNA depleted? Related, it would probably be good to specifically state in the text why rRNA depletion is required to see non-poly-adenylated transcripts.

Figure 5C, D
Given that Figure 5D shows smFISH for ChrIV data, why is that data not shown in the main Figure 5? Related, it would be good to indicate the probe that was used for the smFISH analysis in the genome browser snapshot.

Figure 6 F, G
At what timepoint were the analyses shown in Figures 6F and G done?

Figure 7B and related text
The authors should explain in the main text what a ping pong score is. In the Figure, it is not clear what the y-axis reflects is and how this relates to the ping pong score

Figure 7C
-- means ignored. But what data does this apply to?

Figure 7C, D
D should come before C to show the clusters that are used in C.

Typos:
Line 300 - betweeb
Line 927 – arrowhead and arrowheads…
Line 417 – We PERLs

Reviewer 2

Advance summary and potential significance to field

The manuscript “Extensive nuclear gyration and pervasive non-genic transcription during primordial germ cell development in zebrafish” Redl et al provides a detailed analysis of cell and nucleus shape, expression of RNA and chromatin structure in PGCs during early stages of gonad development. The authors describe the evolution of nuclear shape employing anti-LaminB1 antibody and revise earlier conclusions regarding the subcellular localization of proteins involved in piRNA metabolism. The paper includes a very detailed description of the chromatin state concerning markers for active and inactive domains, a time course of transcription of intragenic domains and analyze PGC-Expressed non-coding RNA Loci (PERLs). Based on the gene expression pattern of germline-specific transcripts, the authors conclude that the commitment to this lineage occurs relatively late, at 3 days post fertilization.

Comments for the author

Main issues-

© 2020. Published by The Company of Biologists under the terms of the Creative Commons Attribution License (https://creativecommons.org/licenses/by/4.0/).

1. The paper includes an impressive amount of data and is thus likely to serve as an important resource in the field of germline development in different organisms. At the same time, the manuscript lacks a hypothesis or functional analysis of the proteins and RNAs reported on. A related issue is that the paper consists of chapters that are sometimes not linked by a specific aim of addressing a specific question. Overall, the paper constitutes an important resource, but is primarily descriptive. The paper would benefit a lot from a demonstration of a use of at least some of the data for understanding some aspects of a known mutant, or from (even preliminary) functional analysis of an RNA or protein identified in this work.
2. An important claim the authors make is that commitment to the germline occurs around 3dpf and not earlier. This supposition is based on gene expression patters and findings concerning germ cell morphology. As such a statement is very important and can direct further investigations in different groups, I believe that it should be substantiated experimentally.

Minor points-
1. For Figure 1- Show Zili subcellular localization at stages later than 3dpf (as for Ziwi).
2. " …nuclear pores were often detected close to nuage (Fig. 2A')" – from the provided panel, it is not clear how the position of the nuclear pores was determined in the EM.
3. Suppl Figure 1 – in E, label the Y-axis properly.
4. The Tdrd6 results "…The Tudor proteins Tdrd1 and Tdrd6a have been shown to be necessary for either nuage or proper germ plasm formation in zebrafish" are presented in the section "Electron microscopy reveals different nuage types during development" where it appears misplaced. Would be better to present these findings elsewhere in the paper.
5. The statement "and found that the level of heterochromatin at 6 dpf was significantly lower than at 1 and 3 dpf (Fig. S1D, E). " …does not fit what is stated later "H3K9me3 staining was relatively low in PGCs between 1 and 6 dpf, and when present it was rather diffuse". This discrepancy should be discussed / corrected.
6. In Figure 3C the ratio between the PGC and somatic cells regarding the level of H3K9 me3 is larger than 1, which is not what one observed in panel A and what is described in the text (…compared to surrounding somatic cells, H3K9me3 staining was relatively low in PGCs between 1 and 6 dpf…).
7. The authors state that the gyrated nuclei are the ones that are transcriptionally active ("…PGC nuclei become extremely gyrated, displaying general broad opening of chromatin and high levels of intergenic transcription."), while the round ones are not, but they state that "…H3K4me3 appeared to be more abundant at 10 dpf, specifically in nuclei with a round morphology ", which seems contradictory.
8. In contrast with the rather general findings concerning morphology and gene expression, the authors present some very specific data regarding splicing of a few genes (Figure 4E,F). The connection of these results to the rest of the paper is very weak, so this information should better be presented in a different publication.
9. Regarding Figure 4B – "Cluster 1 is composed of many genes whose transcripts are known to be maternally provided via the germ plasm, such as granulito, nanos3, tdrd7a and ca15b". This is perhaps consistent with the 1->10dpf data, but is not observed in the Maternal yes/no columns.
10. The information concerning Tdrkh should better not be included in the paper. The authors make some conclusions regarding the function of the protein and connecting it to Redox potential, without showing any data supporting it.
11. In Figure 5B, define the y=axis ("FPKM (+1)").
12. Why was Chromosome 20 chosen for analysis?
13. The authors claim that open chromatin is correlated to an increase in PERLS ("PGC nuclei become extremely gyrated, displaying general broad opening of chromatin and high levels of intergenic transcription. This is accompanied by changes in nuage morphology, expression of large loci (PGC-Expressed non-coding RNA Loci, PERLs)"), but then show that it is over-represented in closed chromatin ("PERLs were over-represented in B compartments.").
14. In Figure 6E, define RPKM for the y-axis.
15. Be consistent with "PERL" between the figures and the text (small letters in the Figures, "Perl" vs "Perls" etc"

© 2020. Published by The Company of Biologists under the terms of the Creative Commons Attribution License (https://creativecommons.org/licenses/by/4.0/).

First revision

Author response to reviewers' comments

We thank the reviewers for their useful comments on our manuscript, and we were happy to read that they value the insights that are gained from this study. A number of valuable suggestions were done to further improve our work, and we have been happy to address these. Unfortunately, we were hampered by the effects that the lockdown in spring has had on our aquarium, hence the delayed re-submission. Nevertheless, we are now in the situation to resubmit a strengthened version of our manuscript. Apart from reviewer-requested changes, we also worked on shortening the text, in order to get within the requested word limit. We sincerely hope that the manuscript is now seen as suitable for publication in Development.

Point-by-point list:

Reviewer 1

…The manuscript contains a wealth of data including the discovery of extensive nuclear gyration in PGCs, changes in nuage morphology, the dynamics of gene expression in PGCs, and the identification of PERLs. These data will be important to further our understanding of germ cell development should be published without a doubt. We do feel, however, that the authors could try to connect the different findings in the paper better (mostly in the text) in order to better explain the advance it provides for the field.

We have improved the transitions between the different sections by including concluding remarks and providing logical steps to the next section.

Reviewer 1 Comments for the Author:
Below, we have listed some minor concerns / suggestions:

Figure 3 (and Figure S1)
Heterochromatin is analyzed by EM (Figure S1D,E) and IF (Figure 3A). The data of these two methods does not result in the same conclusion. A related issue concerns the analysis of the IF data: signal intensity in the PGCs is normalized to signal intensity in the soma. To justify this, the authors should show that the signal in the soma does not change over time. Visual inspection of the images in Figure 3A, however, suggests that this is not the case. Finally, we note that the almost unchanged H3K9me3 signal in PGCs observed does not support claims later in the manuscript that the increase in expression of the kdm4aa demethylase is driving the loss of heterochromatin. The authors should address these issues.

The point raised by the reviewer is well taken, but we believe it stems from a misunderstanding, caused by how we presented the results.
The loss of heterochromatin, as judged by EM is very clear. Basically, this is also what we see in IF. We agree, however, that the IF data is much harder to interpret. Absolute normalization is almost impossible, especially with whole-mount imaging, which creates differences in fluorescence also due to variation in depth of the signal within the sample. We have made several changes to the text to accommodate this. First, we make it very clear that the observations we make are relative to surrounding somatic cells, and add the following text to the results section that describes the IF studies:

“We note that quantification of these experiments is difficult, because the somatic cells that we use for normalization may also display global chromatin changes. However, the relative loss of H3K9me3 in PGCs is consistent with the observed loss of electron-dense chromatin in the EM experiments, and the strong Pol2Ser2P signal starting at 3dpf suggests that transcriptional strongly increases at that point in time. Therefore, we next aimed to analyze the transcriptional output of the PGCs at the different developmental stages.”

Second, we corrected the erroneous suggestion that H3K9me3 staining is low in PGCs at 1dpf. While this may appear as such from the one image, quantification of more images (as we presented) shows it is not much different from the surrounding somatic cells:

© 2020. Published by The Company of Biologists under the terms of the Creative Commons Attribution License (https://creativecommons.org/licenses/by/4.0/).

“First, we analyzed H3K9 tri-methylation (H3K9me3) as a mark associated with heterochromatin and repressed transcription (Penagos-Puig and Furlan-Magaril, 2020). Using the signal from the somatic cells to normalize, we found that H3K9me3 in PGCs dropped between 1dpf and 6dpf (Fig. S3A, B). Additionally, H3K9me3 staining was diffuse in PGCs and only acquired the punctate appearance, which is visible in somatic nuclei, in some PGC nuclei at 10dpf (Fig. S3A, arrow heads).”

Finally, we also adjusted the statement regarding the kdm4aa demethylase. We now write:

“Possibly, expression of this gene could play a role in the observed loss of heterochromatin as seen in EM (Fig. S1) and H3K9me3 stainings (Fig. S3A, B), although this idea needs experimental validation.”

We are of the opinion that this helps to interpret the different gene clusters we describe in the RNAseq section, and/or to start putting that into perspective, but we would have no problem taking this statement out altogether if this is seen as an overstatement.

Figure 3A, legend
Please indicate what panels on the right are, like was done in 3B

This has been done. Note that this Figure now is Figure S3.

Figure 3B, legend
Pol2Ser should be replaced with Pol2Ser2P to indicate the phosphorylation state which is the hallmark of transcription elongation.

This has been done. Note that this Figure now is Figure S4.

Figure 3C
The quantification of signal is at odds with the emphasis on the K9me3 foci. Can the authors explain how they interpret both types of signal?

We specifically mention the H3K9me3 foci, as this is a characteristic property of this mark. However, as we now state more clearly, in PGCs H3K9me3 staining is rather diffuse, and we only start to see foci in some PGCs at 10dpf. In contrast, almost all somatic cells show these foci at all stages. We do not see how this is at odds with the quantification we present. Since foci are basically absent from the PGCs at the quantified timepoints (1 and 6dpf), and always present in somatic cells, they will not affect the analysis a lot. We did add a sentence to the discussion mentioning these foci, but would not like to say anything more specific about them:

“We hypothesize that the answer to the “how” question of PERL activation may lie in a generally increased accessibility of transcription factors to the genome, also to heterochromatic regions. In flies piRNA clusters are heterochromatic, yet they are transcribed by a specialized transcription machinery (ElMaghraby et al., 2019), and also in the fission yeast Schizosaccharomyces pombe, heterochromatin is linked to transcription of small RNA precursors (Keller et al., 2012). By analogy, PERLs may represent such heterochromatic, yet transcribed loci in the zebrafish. Indeed, we do find PERLs enriched in typically ‘closed’ regions of the genome (B compartments), but more detailed analysis of chromatin state will be required to dissect chromatin states in the PGCs at various developmental stages. Such analyses may also provide explanations for the apparent lack of H3K9me3 foci in PGCs until 10dpf.“

The difference between the foci and more diffuse staining may find its origin in 3D organization of chromatin. However, since we do not have any experiments that probe this, we prefer not to speculate on this specific aspect. It certainly is something that we find interesting to follow up on.

Figure S1D
It would be helpful to outline the nuclei that were analyzed.

The nuclei in the relevant panel have been outlined.

© 2020. Published by The Company of Biologists under the terms of the Creative Commons Attribution License (https://creativecommons.org/licenses/by/4.0/).

Figure S1E
How many nuclei were analyzed?

This is now indicated in the figure.

Figure S2
The rationale for the H3K4me2 and H3K4me3 staining and the conclusions drawn from it could be better explained in the text. Is this the 'opening of chromatin' the authors write about in the abstract? Was the specificity of the H3K4me2 Ab tested?

We probed H3K9 and H3K4 methylation as representatives of transcriptionally inactive and active chromatin respectively. We changed the text to make this more clear.

"We observed a change in appearance of chromatin during PGC development in electron micrographs (Fig. S1D, E). This prompted us to investigate chromatin and RNA Polymerase 2 (Pol2) status in PGCs using immune histochemistry. First, we analyzed H3K9 tri-methylation (H3K9me3) as a mark associated with heterochromatin and repressed transcription (Penagos-Puig and Furlan-Magaril, 2020). Using the signal from the somatic cells to normalize, we found that H3K9me3 in PGCs dropped between 1dpf and 6dpf (Fig. S3A, B). Additionally, H3K9me3 staining was diffuse in PGCs and only acquired the punctate appearance, which is visible in somatic nuclei, in some PGC nuclei at 10dpf (Fig. S3A, arrow heads). Second, we analyzed H3K4me2 and H3K4me3, marks associated with transcription (Sims and Reinberg, 2006). These marks did not show strong global dynamics in the PGCs (Fig. S3C, D), although some PGC nuclei showed stronger H3K4me3 signal at 6 and 10dpf (Fig. S3D)."

We did not test the specificity of the antibodies we used ourselves. The specific antibodies we used are now indicated in the Materials and Methods section (apologies for that omission), and these have been used in other studies as specific markers of, and for ChIP experiments on the relevant histone modifications.

Figure 4A, B
The color codes used for timepoints in panels A and B is different which is confusing.

The color code has been made consistent.

Figure 4B and related text
Inconsistent use of cluster/group in the text (cluster 1, cluster 2, group 3, group 4).

This has been corrected.

Figure 5 and related text
The authors write "The zygote was almost entirely deprived of intergenic transcripts, as expected by the lack of zygotic transcription". These reviewers does not understand this rationale. Could intergenic transcripts not be maternally loaded?

The phrasing was not accurate. This paragraph has been adjusted to more accurately convey what we meant:

"Next, we analyzed rRNA-depleted libraries (referred to as "total RNA") from the same time points. As rRNA is very abundant, its depletion is needed to reliably detect non-poly-adenylated transcripts, such as for instance intergenic transcripts. The zygote was almost entirely deprived of intergenic transcripts (Fig. 5A), and 1, 2 and 3dpf slight increases in intergenic transcription in both PGCs and 'whole fish' samples were detected. At 6dpf a striking increase in intergenic expression was observed only in the PGCs, and this further increases at 10dpf. This increase in intergenic transcription is not an artefact caused by the increase of just a few loci, which could represent non-annotated genes, since the number of intergenic loci in PGCs that show this trend also increases over time (Fig. 5B, S5A). We conclude that intergenic transcription is strongly activated in PGCs, starting between 3 and 6dpf."

© 2020. Published by The Company of Biologists under the terms of the Creative Commons Attribution License (https://creativecommons.org/licenses/by/4.0/).

Figure 5A
This is not total RNA but rRNA depleted? Related, it would probably be good to specifically state in the text why rRNA depletion is required to see non-poly-adenylated transcripts.

The adjusted paragraph cited above also addresses this issue.

Figure 5C, D
Given that Figure 5D shows smFISH for ChrIV data, why is that data not shown in the main Figure 5? Related, it would be good to indicate the probe that was used for the smFISH analysis in the genome browser snapshot.

This has been adapted as suggested. We note in the legend that the used probe in fact does not overlap with a called PERL. We designed the probe-set before we had called the PERLS, and this region is apparently missed by our annotation criteria. We have tried to find additional good sites for probe design but the highly repetitive nature of these regions makes it very hard to design more selective probe-sets.

Figure 6 F, G
At what timepoint were the analyses shown in Figures 6F and G done?

The PERLs are called using RNA expression from the different time points, and as a result the PERLs are themselves not time-point dependent. After we called them, they are simply annotated regions within the genome, just like for instance transposon annotations. Hence, the panels 6F and 6G are also not time-point dependent; they reflect what fraction of the PERLs overlaps with transposons.

Figure 7B and related text
The authors should explain in the main text what a ping pong score is. In the Figure, it is not clear what the y-axis reflects is and how this relates to the ping pong score

We explain in the introduction what a ping-pong signature is. However, as this is a rather field-specific term, we again briefly explain it now in the results section. We also improved the legend. We hope this provides significant clarity for research from outside the piRNA-field:

“Interestingly, at 6dpf this anti-sense bias started to decrease significantly (Fig. 7A), accompanied by an increase in ping-pong signature: the characteristic enrichment of 10 base pair overlap of the 5’ ends of piRNAs, caused by the nuclease properties of Piwi proteins (Fig. 7B). These results, combined with the above described increase in transcription of piRNA pathway components, are clear indications that between 3 and 6dpf the piRNA pool starts to shift from purely maternally derived material to zygotically produced piRNAs.”

Figure 7C
-- means ignored. But what data does this apply to?

This was automatically generated by the plotting library, but is meaningless for our plot. We have removed it.

Figure 7C, D
D should come before C to show the clusters that are used in C.

This has been corrected.

Typos:
Line 300 - betweeb
Line 927 – arrowhead and arrowheads… Line 417 – We PERLs

These have been corrected.

© 2020. Published by The Company of Biologists under the terms of the Creative Commons Attribution License (https://creativecommons.org/licenses/by/4.0/).

Reviewer 2

Main issues-
1. The paper includes an impressive amount of data and is thus likely to serve as an important resource in the field of germline development in different organisms. At the same time, the manuscript lacks a hypothesis or functional analysis of the proteins and RNAs reported on. A related issue is that the paper consists of chapters that are sometimes not linked by a specific aim of addressing a specific question. Overall, the paper constitutes an important resource, but is primarily descriptive. The paper would benefit a lot from a demonstration of a use of at least some of the data for understanding some aspects of a known mutant, or from (even preliminary) functional analysis of an RNA or protein identified in this work.

We agree that our manuscript is descriptive, but we would like to stress that the lack of description of these stages significantly hampers the zebrafish germ cell field. The reason for why these developmental steps had not yet been described likely is that the material is rather limited, and the cells are hard to see in the embryo.
Combined with the idea that these cells are ‘resting’ (something that was again mentioned during the latest germ cell meeting), this likely led to a lack of interest to study these cells between 1 and 10dpf. Focusing the attention on these stages by a descriptive paper is therefore very important.

Even if nowadays more and more can be done with less and less cells, manipulation of these cells at the required timepoints is far from trivial. This is also connected to the fact that before cell sorting, embryos will need to be genotyped. This combination currently makes most mutant analyses hard, and not feasible in a revision period.
Hence, we cannot provide mutant data related to our genomics experiments. However, we have added new EM data in a new Figure 3 and S2, showing that the Piwi pathway is crucial in achieving the switch in nuage morphology that we describe:

“Transition of granular to compact nuage requires zygotic Tdrd1 and Zili
The Tudor proteins Tdrd1 and Tdrd6a have been shown to be necessary for either nuage or proper germ plasm formation in zebrafish (Huang et al., 2011; Roovers et al., 2018). In *tdrd6a* mutants embryonic germ plasm is affected, but nuage seems unaffected (Roovers et al., 2018). Here, we probed the presence and localization of Tdrd6a in 1, 3, 6 and 10dpf-old PGCs (Fig. S1F). Tdrd6a colocalized with Ziwi in large perinuclear nuage granules at 1dpf, but in 3dpf PGCs was found either colocalizing with Ziwi in perinuclear nuage or was absent from PGCs (* in Fig. S1F, 3dpf). At 6dpf, Tdrd6a protein was no longer detected in PGCs, and only at 10dpf some germ cells showed re-appearance of Tdrd6a protein (Fig. S1F). We hypothesize that cells with Tdrd6a correspond to cells displaying granular nuage in EM, and those lacking Tdrd6a to cells displaying adult-type nuage. Tdrd1 is only expressed zygotically, starting around 3dpf (Huang et al., 2011), coinciding with the emergence of compact nuage (Fig. 2B). In adults, Tdrd1 is found in the compact nuage that is typical for adult germ cells, and in *tdrd1* mutant adult germ cells no nuage can be detected (Huang et al., 2011). We tested the effect of Tdrd1 on the described nuage dynamics in PGCs using EM. As in the adult, *tdrd1* mutant PGCs at 6dpf did not have detectable compact nuage (Fig. 3A, B, S2). However, these PGCs did contain the granular nuage that is typical of 1-2dpf PGCs (Fig. 2A, B).
We also tested if Zili, which like Tdrd1 only starts to be expressed at 3dpf (Houwing et al., 2008), is required for this shift in nuage morphology. Indeed, loss of Zili led to a very similar phenotype at 6dpf, where only granular nuage and no compact nuage can be detected (Fig. 3C, S2). Interestingly, we did observe patches of darker nuage in some cells (Fig. S2), as we also observed in some granular nuage patches at 3dpf in wild-type animals (Fig. 2F). In contrast, homozygous *ziwi* mutant PGCs did not show this phenotype, and contained dark, adult-like nuage (Fig. 3C, S2). This lack of a phenotype likely stems from maternal Ziwi deposition (Houwing et al., 2007). Overall, these results show that the piRNA pathway is an important factor behind the observed changes in nuage morphology in germ cells, and that different Tudor-domain proteins mark different types of nuage.”

We believe that this achieves what the reviewer requests: a sign that at least some of the observations can be linked to concrete molecular pathways. We, and hopefully inspired by this paper also others, intend to delve into much more details using ChIPseq, ATACseq etc., and to

© 2020. Published by The Company of Biologists under the terms of the Creative Commons Attribution License (https://creativecommons.org/licenses/by/4.0/).

follow up on a number of interesting PGC-expressed genes, but these are complete PhD trajectories on their own.

2. An important claim the authors make is that commitment to the germline occurs around 3dpf and not earlier. This supposition is based on gene expression patters and findings concerning germ cell morphology. As such a statement is very important and can direct further investigations in different groups, I believe that it should be substantiated experimentally.

We purposely phrased the possibility of late germ cell fate establishment very weak. In the results section:
"It is possible that PGCs only become fully committed germ cells once they interact with the cells at the genital ridge at this point in development and that the cluster 4 genes play a role in this process."

And in the Discussion section:
"Possibly, these somatic cells represent niches from which the PGCs receive signals that trigger their further development."

To study these ideas further would require extensive experiments, including the generation of mutant lines, germ cell transplantations etc. Clearly, that is beyond the scope of this manuscript. We did attempt to verify the *prdm1b* expression using smFISH, but despite several attempts, we were not able to obtain specific signal for *prdm1b*, or *vasa* (as control) at later timepoints. Such orthogonal verification of the expression of such a gene would have been nice, but we feel that it is not crucial for the manuscript, and we feel that this minor problem should not delay its publication any further.

Minor points-
1. For Figure 1- Show Zili subcellular localization at stages later than 3dpf (as for Ziwi).

Added as Fig. 1E.

2. " …nuclear pores were often detected close to nuage (Fig. 2A')" – from the provided panel, it is not clear how the position of the nuclear pores was determined in the EM.

We appreciate that the detection of nuclear pores is not always trivial. They are typically visible as dark patches within, or interrupting the nuclear envelope. We now show a clearer example of nuage and nuclear pores at 1dpf, and also tried to clarify it better in the legend to Figure 2. We hope the reviewer can better appreciate the nuclear pore-nuage connection with this new image.

3. Suppl Figure 1 – in E, label the Y-axis properly.

We have better annotated the Y axis in the indicated figure, and also improved the text of the corresponding legend.

4. The Tdrd6 results "…The Tudor proteins Tdrd1 and Tdrd6a have been shown to be necessary for either nuage or proper germ plasm formation in zebrafish" are presented in the section "Electron microscopy reveals different nuage types during development" where it appears misplaced. Would be better to present these findings elsewhere in the paper.

We have given this section its own title, and, as described above, have expanded its contents with *tdrd1* and *zili*
mutant data (Figures 3 and S2 in the new manuscript).

5. The statement "and found that the level of heterochromatin at 6 dpf was significantly lower than at 1 and 3 dpf (Fig. S1D, E). " …does not fit what is stated later "H3K9me3 staining was relatively low in PGCs between 1 and 6 dpf, and when present it was rather diffuse". This discrepancy should be discussed / corrected.

In EM we observe a loss of dark nuclear patches, which are characteristic of heterochromatin, during PGC development. Using IF, we see that H3K9me3 drops in between 1 and 6dpf. We believe

© 2020. Published by The Company of Biologists under the terms of the Creative Commons Attribution License (https://creativecommons.org/licenses/by/4.0/).

these are findings that are not in conflict with each other. At 1dpf, indeed the statement with regard to somatic cells (see point 6.) was not correct; quantification of multiple images showed that H3K9me3 staining at 1dpf is not weaker than in the soma. To better reflect the data we rephrased the text as follows:

"First, we analyzed H3K9 tri-methylation (H3K9me3) as a mark associated with heterochromatin and repressed transcription (Penagos-Puig and Furlan-Magaril, 2020). Using the signal from the somatic cells to normalize, we found that H3K9me3 in PGCs dropped between 1dpf and 6dpf (Fig. S3A, B). Additionally, H3K9me3 staining was diffuse in PGCs and only acquired the punctate appearance, which is visible in somatic nuclei, in some PGC nuclei at 10dpf (Fig. S3A, arrow heads). Second, we analyzed H3K4me2 and H3K4me3, marks associated with transcription (Sims and Reinberg, 2006). These marks did not show strong global dynamics in the PGCs (Fig. S3C, D), although some PGC nuclei showed stronger H3K4me3 signal at 6 and 10dpf (Fig. S3D). Finally, we addressed Pol2 activity by staining for the elongation-specific Ser2P modification (Fig. S4A). PGCs at 1dpf had levels of Pol2Ser2P that are similar to the levels observed in their somatic neighbors. Starting in some cells at 3dpf (Fig. S4A; right-most cell; S4B), and in all cells at 6dpf, we observed increasing Pol2 elongation activity in PGC nuclei compared to that in the neighboring somatic nuclei. At 10dpf some cells still showed high Pol2Ser2P levels, whereas others returned to lower levels (Fig. S4A)."

6. In Figure 3C the ratio between the PGC and somatic cells regarding the level of H3K9 me3 is larger than 1, which is not what one observed in panel A and what is described in the text (…compared to surrounding somatic cells, H3K9me3 staining was relatively low in PGCs between 1 and 6 dpf…).

Please see previous point. The changed text in the results section takes care of the unintended apparent discrepancy.

7. The authors state that the gyrated nuclei are the ones that are transcriptionally active ("…PGC nuclei become extremely gyrated, displaying general broad opening of chromatin and high levels of intergenic transcription."), while the round ones are not, but they state that "…H3K4me3 appeared to be more abundant at 10 dpf, specifically in nuclei with a round morphology ", which seems contradictory.

We did not intent to convey the message that only the gyrated nuclei are transcriptionally active. In fact, we show that many genes are expressed at any time point that we analyzed. We hypothesize that the gyrated nuclei are connected to the transcriptional activation of the PERLs (Discussion). However, we do not know anything about the chromatin status of the PERLs during their transcription, so how this would relate to H3K4 methylation is currently unclear. It is clear that the chromatin analysis we present is very descriptive, and for more detailed statements ChIPseq related experiments will be required. Given the very low cell numbers we can isolate, this has thus far not been possible. We make this point explicitly now in the results section that describes the IF experiments, in order to prevent the possible impression of overinterpretation of data that is inherently difficult to quantify:

"We note that quantification of these experiments is difficult, because the somatic cells that we use for normalization may also display global chromatin changes. However, the relative loss of H3K9me3 in PGCs is consistent with the observed loss of electron-dense chromatin in the EM experiments, and the strong Pol2Ser2P signal starting at 3dpf suggests that transcriptional strongly increases at that point in time."

8. In contrast with the rather general findings concerning morphology and gene expression, the authors present some very specific data regarding splicing of a few genes (Figure 4E,F). The connection of these results to the rest of the paper is very weak, so this information should better be presented in a different publication.

This analysis has been deleted.

9. Regarding Figure 4B – "Cluster 1 is composed of many genes whose transcripts are known to be maternally provided via the germ plasm, such as granulito, nanos3, tdrd7a and ca15b". This is

© 2020. Published by The Company of Biologists under the terms of the Creative Commons Attribution License (https://creativecommons.org/licenses/by/4.0/).

perhaps consistent with the 1-
>10dpf data, but is not observed in the Maternal yes/no columns.

The genes labelled as 'Maternal' were identified previously as transcripts that are loaded into the embryo. This does not imply that they do not become transcribed at later time points as well, and that at these later time points they may in fact be much higher expressed. All these such dynamics feed into the clustering of the genes in our analysis, and hence, 'Maternal' transcripts can be found in all four clusters.

10. The information concerning Tdrkh should better not be included in the paper. The authors make some conclusions regarding the function of the protein and connecting it to Redox potential, without showing any data supporting it.

We agree this was very speculative, and have deleted this section and figure from the manuscript.

11. In Figure 5B, define the y=axis ("FPKM (+1)").

Done.

12. Why was Chromosome 20 chosen for analysis?

The displayed chromosomes happen to have PERLs that are very nicely visible, either due to their size and/or their clustering. We show in Fig. 6 that this is no special characteristic of any one chromosome (with the exception of chr. 4, which is very rich in PERLs).

13. The authors claim that open chromatin is correlated to an increase in PERLS ("PGC nuclei become extremely gyrated, displaying general broad opening of chromatin and high levels of intergenic transcription. This is accompanied by changes in nuage morphology, expression of large loci (PGC-Expressed non-coding RNA Loci, PERLs)"), but then show that it is over-represented in closed chromatin ("PERLs were over-represented in B compartments.").

This is correct, and in fact is an interesting point. We address this now more explicitly in the discussion. I short: piRNA clusters in flies are known to be transcribed, despite being in a heterochromatic state, and also in fission yeast, heterochromatin is linked to transcription of small RNA precursors. Since PERLs show similarities to piRNA clusters we hypothesize that they are transcribed in a similar manner, from heterochromatin. The text in the discussion has been adapted:

"We hypothesize that the answer to the "how" question of PERL activation may lie in a generally increased accessibility of transcription factors to the genome, also to heterochromatic regions. In flies piRNA clusters are heterochromatic, yet they are transcribed by a specialized transcription machinery (ElMaghraby et al., 2019), and also in the fission yeast Schizosaccharomyces pombe, heterochromatin is linked to transcription of small RNA precursors (Keller et al., 2012) . By analogy, PERLs may represent such heterochromatic, yet transcribed loci in the zebrafish. Indeed, we do find PERLs enriched in typically 'closed' regions of the genome (B compartments), but more detailed analysis of chromatin state will be required to dissect chromatin states in the PGCs at various developmental stages. Such analyses may also provide explanations for the apparent lack of H3K9me3 foci in PGCs until 10dpf."

14. In Figure 6E, define RPKM for the y-axis.

Done.

15. Be consistent with "PERL" between the figures and the text (small letters in the Figures, "Perl" vs "Perls" etc"

Done.

© 2020. Published by The Company of Biologists under the terms of the Creative Commons Attribution License (https://creativecommons.org/licenses/by/4.0/).

Second decision letter

MS ID#: DEVELOP/2020/193060

MS TITLE: Extensive nuclear gyration and pervasive non-genic transcription during primordial germ cell development in zebrafish

AUTHORS: Stefan Redl, Antonio Miguel de Jesus Domingues, Edoardo Caspani, Stefanie Moeckel, Willi Salvenmoser, Maria Mendez-Lago, and Rene Ketting

I have received all the reviewer comments, and as you will see the overall evaluation is positive and we would like to publish a revised manuscript in Development. However, both reviewers highlight some textual edits which will greatly improve the clarity of the manuscript. Please attend to these suggestions and detail them in your point-by-point response. I do not expect to send the study back to the reviewers, however, it is important that you make the suggested edits. Please highlight all the textual changes in the revised manuscript. The referees' comments are appended below, or you can access them online: please go to BenchPress and click on the 'Manuscripts with Decisions' queue in the Author Area.

Reviewer 1

Advance summary and potential significance to field

We think that the revised version of this manuscript is well-suited to be published in Development. Only two minor comments on the text remain.

Comments for the author

Minor comments on text.

Lines 183-185 In “We conclude that PGCs undergo many morphological changes between 1 and 6dpf, showing that these cells are not resting. We next analyzed these two aspects in further detail, starting with nuage dynamics.” it is not clear which 2 aspects the authors are referring to.

Line 379 --
While it is noted that the focus of the hierarchical clustering of TE-derived piRNAs (Fig 7C) is on cluster 5, perhaps a sentence can be added to summarize what characterizes the other clusters shown in the figure?

Reviewer 2

Advance summary and potential significance to field

The paper provides a large amount of information concerning the development of germ cells within the early developing gonad.
It will constitute an important resource that will have a strong impact on studies in zebrafish and in other organisms.

Comments for the author

The authors revised the paper extensively and the current version should be published in my opinion, pending very minor corrections listed below-

1. "...whereas Zili is both in the cytoplasm as well as within the nucleus at 3-7dpf, but becomes restricted to perinuclear granules at later stages." - provide a reference supporting this statement.

© 2020. Published by The Company of Biologists under the terms of the Creative Commons Attribution License (https://creativecommons.org/licenses/by/4.0/).

2. "However, at 3 and 6dpf we found the nuclear envelope to be heavily gyrated.." - in Figure 1 the "6dpf" is missing.

3. "...both cytoplasm and chromatin at 3dpf (Fig. 1C,D), when Zili starts..." - Panel D is not labelled in the Figure.

4. "...dark staining patches all along the nuclear envelope, very similar in appearance to nuage found in adult germ cells (Fig. 2C)." - cite Fig 2C after "envelope" and not at the end of the sentence. As it is, one expects to see in 2C adult germ cells.

5. "...between 1 and 6dpf, showing that these cells are not resting." - remove the "showing that these cells are not resting". Not clear what "resting" means exactly and it is not needed to state that.

6. "As in the adult, tdrd1 mutant PGCs at 6dpf did not have detectable compact nuage (Fig. 3A, B, S2)." - In the tdrd1-/-, just below the left magnification box there seem to be compact nuage.

7. "Starting in some cells at 3dpf (Fig. S4A; right-most cell; S4B),..." - use a comma after "S4A" not a semicolon.

8. "It is known that PGCs in zebrafish lose their germ cell fate if they do not reach the genital ridge (Gross-Thebing et al., 2017)" - in fact, this paper shows the opposite for non-manipulated cells.

Second revision

Author response to reviewers' comments

General comments:

Apart from the changes requested by the reviewers (see below) we also added a phrase to the legend of Figure S2, as we noticed a discontinuity in one of the images displayed there. This results from image acquisition, and not from image manipulation. We prefer to explain this in the legend, rather than to try and fix this visually:
We note that the second image of the zili mutant contains a horizontal break in continuity approximately at the middle of the image. This is the result of image acquisition of larger images, where software needs to stich multiple images together. This process is sometimes not perfect and can create artefacts at the overlap.

Also, we refitted our main Figures to fit the 180x210mm requirement, This led to a rearrangement in Figures 4 and 6, but nothing was changed to the contents of the Figures.

Response to reviewer comments

Reviewer 1 Comments for the Author:
Minor comments on text.

Lines 183-185
In "We conclude that PGCs undergo many morphological changes between 1 and 6dpf, showing that these cells are not resting. We next analyzed these two aspects in further detail, starting with nuage dynamics." it is not clear which 2 aspects the authors are referring to.

Response:
These two sentences have been replaced with:
We conclude that PGCs undergo many morphological changes between 1 and 6dpf. Next we addressed the requirement of the piRNA pathway in the described nuage changes specifically.

© 2020. Published by The Company of Biologists under the terms of the Creative Commons Attribution License (https://creativecommons.org/licenses/by/4.0/).

Line 379 --
While it is noted that the focus of the hierarchical clustering of TE-derived piRNAs (Fig 7C) is on cluster 5, perhaps a sentence can be added to summarize what characterizes the other clusters shown in the figure?

Response:
The following sentence has been added:
Interestingly, piRNAs from other TE clusters, including DNA transposons, endogenous retroviruses but also LTR retrotransposons are enriched at earlier timepoints, pointing at potential differential activation of different transposons in the course of development.

Reviewer 2 Comments for the Author:
The authors revised the paper extensively and the current version should be published in my opinion, pending very minor corrections listed below-

1. "...whereas Zili is both in the cytoplasm as well as within the nucleus at 3-7dpf, but becomes restricted to perinuclear granules at later stages." - provide a reference supporting this statement.

Response:

Citation to Houwing et al 2008 has been added.

2. "However, at 3 and 6dpf we found the nuclear envelope to be heavily gyrated.." - in Figure 1 the "6dpf" is missing.

Response:
The label 6dpf somehow got lost in file conversion. It is there in our illustrator file. We made new pdf files of all figures to correct this problem. In addition we resized the figures, and rearranged some of them to match what is requested by Development.

3. "...both cytoplasm and chromatin at 3dpf (Fig. 1C,D), when Zili starts..." - Panel D is not labelled in the Figure.

Response:
The label 'D' somehow got lost in file conversion. It is there in our illustrator file. We made new pdf files of all figures to correct this problem. In addition we resized the figures, and rearranged some of them to match what is requested by Development.

4. "...dark staining patches all along the nuclear envelope, very similar in appearance to nuage found in adult germ cells (Fig. 2C)." - cite Fig 2C after "envelope" and not at the end of the sentence. As it is, one expects to see in 2C adult germ cells.

Response:
Done.

5. "...between 1 and 6dpf, showing that these cells are not resting." - remove the "showing that these cells are not resting". Not clear what "resting" means exactly and it is not needed to state that.

Response:
This has been rephrased:
We conclude that PGCs undergo many morphological changes between 1 and 6dpf. Next we addressed the requirement of the piRNA pathway in the described nuage changes specifically.

6. "As in the adult, tdrd1 mutant PGCs at 6dpf did not have detectable compact nuage (Fig. 3A, B, S2)." - In the tdrd1-/-, just below the left magnification box there seem to be compact nuage.

Response:
There are dark patches there, but these are have a membrane (enclosed by a sharp line) and most likely are multi-vesicular bodies. We indicate these now with an asterisk and explain that in the

© 2020. Published by The Company of Biologists under the terms of the Creative Commons Attribution License (https://creativecommons.org/licenses/by/4.0/).

legend:
Asterisk: multi-vesicular-bodies, not compact nuage.

7. "Starting in some cells at 3dpf (Fig. S4A; right-most cell; S4B),..." - use a comma after "S4A" not a semicolon.

Response:

Corrected.

8. "It is known that PGCs in zebrafish lose their germ cell fate if they do not reach the genital ridge (Gross-Thebing et al., 2017)" - in fact, this paper shows the opposite for non-manipulated cells.

Response:
We have rephrased this to:
It is known that PGCs in zebrafish **can** lose their germ cell fate…
A more extensive discussion of this would require too much space in relation to the data that are behind this statement.

Third decision letter

MS ID#: DEVELOP/2020/193060

MS TITLE: Extensive nuclear gyration and pervasive non-genic transcription during primordial germ cell development in zebrafish

AUTHORS: Stefan Redl, Antonio Miguel de Jesus Domingues, Edoardo Caspani, Stefanie Moeckel, Willi Salvenmoser, Maria Mendez-Lago, and Rene Ketting
ARTICLE TYPE: Research Article

I am happy to tell you that your manuscript has been accepted for publication in Development, pending our standard ethics checks.

© 2020. Published by The Company of Biologists under the terms of the Creative Commons Attribution License (https://creativecommons.org/licenses/by/4.0/).
